# Effects of AntagomiRs on Different Lung Diseases in Human, Cellular, and Animal Models

**DOI:** 10.3390/ijms20163938

**Published:** 2019-08-13

**Authors:** Giuseppe Murdaca, Alessandro Tonacci, Simone Negrini, Monica Greco, Matteo Borro, Francesco Puppo, Sebastiano Gangemi

**Affiliations:** 1 Clinical Immunology Unit, Department of Internal Medicine, University of Genoa and Ospedale Policlinico San Martino, 16132 Genoa, Italy; 2 Clinical Physiology Institute, National Research Council of Italy (IFC-CNR), 56124 Pisa, Italy; 3 School and Operative Unit of Allergy and Clinical Immunology, Department of Clinical and Experimental Medicine, University of Messina, 98125 Messina, Italy

**Keywords:** antagomiR, miRNAs, lung diseases, human models, cellular models, animal models

## Abstract

Introduction: MiRNAs have been shown to play a crucial role among lung cancer, pulmonary fibrosis, tuberculosis (TBC) infection, and bronchial hypersensitivity, thus including chronic obstructive pulmonary disease (COPD) and asthma. The oncogenic effect of several miRNAs has been recently ruled out. In order to act on miRNAs turnover, antagomiRs have been developed. Materials and methods: The systematic review was conducted under the PRISMA guidelines (registration number is: CRD42019134173). The PubMed database was searched between 1 January 2000 and 30 April 2019 under the following search strategy: (((antagomiR) OR (mirna antagonists) OR (mirna antagonist)) AND ((lung[MeSH Terms]) OR (“lung diseases”[MeSH Terms]))). We included original articles, published in English, whereas exclusion criteria included reviews, meta-analyses, single case reports, and studies published in a language other than English. Results and Conclusions: A total of 68 articles matching the inclusion criteria were retrieved. Overall, the use of antagomiR was seen to be efficient in downregulating the specific miRNA they are conceived for. The usefulness of antagomiRs was demonstrated in humans, animal models, and cell lines. To our best knowledge, this is the first article to encompass evidence regarding miRNAs and their respective antagomiRs in the lung, in order to provide readers a comprehensive review upon major lung disorders.

## 1. Introduction

MicroRNAs (miRNAs) are small molecules made of 21 nucleotides, which modulate several biological processes through post-transcriptional gene expression regulation [1]. In addition, many miRNA knockout strains have differential responses to models of several disorders such as neuronal, cardiac, pulmonary, vascular, renal, immunological, while some of them have altered susceptibility to fungal or bacterial infections, or altered propensity to develop tumors in cancer models [2]. In fact, miRNAs may control tumor development, both acting as tumor-promoting miRNAs (oncomiRNAs and metastamiRNAs) or as tumor suppressor miRNAs [3,4]. Furthermore, it was demonstrated that several human miRNAs are located in specific genomic sites which are involved in cancer [5]. During the last decade, researchers investigated miRNAs functioning and regulation. More recently, they focused on miRNA stability and turnover. Indeed, it was established that miRNAs are very dynamic molecules, presenting with a rapid turnover, which depends on their activation. On these bases, researchers investigated miRNA-induced silencing complex (miRISC) and Argonaute (AGO) proteins, which directly interact with miRNAs and are key factors in the assembly and function of miRISCs [1]. In order to act on miRNAs turnover, antagomiRs have been developed. They are a novel class of chemically engineered oligonucleotides, which are specific silencers of endogenous miRNAs. Specifically, two major molecular changings have been developed in order to increase chemical stability. Thus, including switching of the phosphodiester support with a phosphorothioate linkage between nucleotides or including a 2′O-methyl group. Additionally, antagomiRs with a cholesterol moiety are thought to promote cellular uptake [6]. Considering the complex role of miRNAs, these new molecules can be powerful tools to silence specific miRNAs in vivo and may represent a therapeutic strategy for silencing miRNAs in disease. Indeed, Krützfeldt et al. [7] conducted a study on mice in order to study the biological significance of miR-122, which is abundant in the liver. Analysis of gene expression of messenger RNA from antagomiR-treated animals disclosed that the 3′ untranslated areas of upregulated genes were extremely enriched with miR-122 recognition motives, while down-regulated genes were poor of these motives. Moreover, researchers found that cholesterol biosynthesis genes would have been modulated by miR-122; in fact, plasma cholesterol levels were reduced in antagomiR-122-treated mice. So far, evidence on antagomiR function has been collected on cellular, animal, and human models. Moreover, as described below, data in these three groups are often coherent. This in support of the hypothesis that miRNAs are molecular factors capable of influencing the expression of several disorders. To date, new studies regarding the use of miRNAs as therapeutic targets are ongoing, especially in the treatment of HCV infection, atherosclerosis, and oncologic diseases. As Gambari et al. [3] highlighted in their review, until now, several antagomiRs have been studied among oncologic diseases as therapeutic agents, both alone or in combination with standard drugs. The promising results explain the reason why these agents will improve the therapy of several tumors, such as gastric cancer, gliomas, and breast cancer [8,9,10]. Indeed, the role of miRNAs upon lung disorders has been extensively studied. MiRNAs have proven to play a crucial role among lung cancer, pulmonary fibrosis, tuberculosis (TBC) infection, and bronchial hypersensitivity, thus including chronic obstructive pulmonary disease (COPD) and asthma. The oncogenic effect of several miRNAs has been recently ruled out. Herein, a study of our group recently identified for the first time new mechanisms, supporting the crucial role of cigarette smoke-induced miR-21 expression in the amplification of inflammatory responses and in tumorigenesis processes within the airways [11]. COPD is a complex disease with a high rate of morbidity and mortality, especially in Western countries. Disease exacerbations and the associated hospitalizations often represent a considerable expense at a socio-economic level. Reduced lung function predicts mortality and is key to the diagnosis of COPD. Shrine et al. [12] conducted a genome-wide associated study in order to highlight new genetic mechanisms in order to improve future preventive and therapeutic strategies for COPD. More recently, many authors investigated miRNAs expression in COPD, noticing that wide networks composed of miRNA and messenger RNA (mRNAs) cooperate in COPD pathogenesis [13]. Moreover, Faiz et al. [14] investigated whether miRNA expression was modulated by inhaled corticosteroid (ICS) treatment and identified miR-320d as a novel mediator of ICS, regulating the pro-inflammatory response of the airway epithelium. Nowadays, among chronic inflammatory lung diseases, asthma is one of the most prevalent. Pathological mechanisms rely on activation of mast cells and eosinophils and dysregulation of Th 2 response. Asthmatic patients often have a strong genetic background, however recent studies demonstrated the role of epigenetic factors such as miRNAs [15]. Gold standard therapies for asthma control include inhaled β-agonists, both short and long acting, and steroids. On this background, Yu at al. [16] recently demonstrated that a specific miRNA, miR-16, may be used as a predictive biomarker of therapeutic response in asthma, thus, suggesting the role of miRNAs not only upon disease physiopathology but also upon drug response. MiRNAs have shown to have an effect also upon pulmonary arterial hypertension (PAH). PAH is a complex disease with different clinical manifestations and high genetic variability. ESC/ERS Guidelines outlined some of the most frequently involved genetic factors such as nuclear factor of activated T cells (NFAT), hypoxia-inducible factor 1α (HIF-1α), and signal transducer and activator of transcription 3 (STAT3) [17]. New research showed that miRNAs may play a crucial role in vascular remodeling, thus inhibiting or promoting pulmonary vascular resistance. Novel genetic studies have not only focused on chronic lung diseases. Acute respiratory distress syndrome (ARDS) and acute lung injury (ALI) are well defined clinical disorders caused by many clinical insults to the lung or because of predispositions to lung injury [18]. These conditions are characterized by massive lung inflammatory response and alveolar barrier damage, and supportive care combined with anti-inflammatory drugs and fluid replacement is fundamental. Currently, there is significant evidence endorsing the crucial role of miRNAs as a new class of gene regulators in ALI [19]. Although several goals have been reached among oncologic disorders, lung cancer still represents a high mortality disease. New discoveries on genetic factors and molecular pathways involved in the disease pathogenesis have been ruled out, however further steps are needed. Indeed, during the last years, miRNAs were found to be useful screening tools. Moreover, they can help clinicians to discriminate between primary lung tumors and lung metastases [20]. Finally, some studies demonstrated that specific miRNAs expression may predict lung tumor prognosis [21]. Most importantly, anti-miRNAs molecules, as antagomiRs, are now emerging as new therapeutic tools. Therefore, the goal of the actual research is to use miRNAs as therapeutic agents. Single miRNAs modulate various mRNA target expressions and may have wide impacts on various cellular processes. Therapies that target individual miRNAs can therefore have wider effects than traditional single-molecule/single-target methods. Indeed, changing numerous downstream objectives, miRNAs may improve the probability of adverse effects occurrence, especially when systemic drug delivery is used. Considering all these, biopharmaceutical companies are actually trying to use miRNAs as novel therapeutics. Indeed, two clinical studies have been launched for hepatitis C virus infection and advanced hepatocellular carcinoma [22,23] treatment. Nevertheless, no clinical trials have yet evaluated the impact on respiratory diseases of miRNA-targeted strategies. To our best knowledge, this is the first article to encompass evidence regarding miRNAs and their respective antagomiRs in the lung. In fact, several authors focused on miRNAs and their blocking agents with respect to different lung diseases such as asthma, COPD, and ARDS. Our work is the first to consider these molecules from a more extensive view, basing on the idea that several miRNAs play a crucial role in different respiratory disorders, thus sharing common biological processes in the lung. Moreover, our research included studies conducted on humans, animals, and cells. The aim of this three-level research was to confirm data from previous analysis and look for a correlation between these three categories. Thus, the idea of lung-specific miRNAs could be hypothesized. Furthermore, this concept would have impact not only on miRNAs knowledge, but also on antagomiR development. New molecules could be tested as more tailored therapeutic agents with less adverse effects.

## 2. Materials and Methods

The systematic review was conducted under the PRISMA guidelines (registration number is: CRD42019134173). The PubMed database was searched between 1 January 2000 and 30 April 2019 under the following search strategy: (((antagomiR) OR (mirna antagonists) OR (mirna antagonist)) AND ((lung[MeSH Terms]) OR (“lung diseases”[MeSH Terms]))). In this analysis, we included original articles, published in English, whereas exclusion criteria included reviews, meta-analyses, single case reports, and studies published in a language other than English [3,7,24,25,26,27,28,29,30,31,32,33,34,35,36,37,38,39,40,41,42,43,44,45,46,47,48,49,50,51,52,53,54,55,56,57,58,59,60,61,62,63,64,65,66,67,68,69,70,71].

## 3. Results

**Figure 1:**
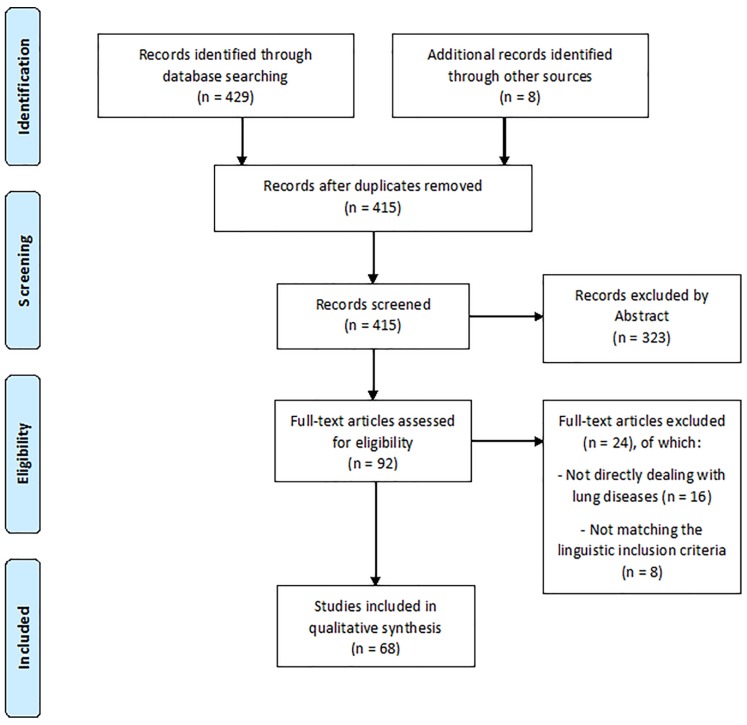
Study selection.

According to the procedure previously described, we retrieved 68 articles matching the inclusion criteria (see the flowchart in Figure 1).

As shown in Table 1, Table 2, Table 3, Table 4 and Table 5, the vast majority of them included research carried out on cell lines and on animals, whereas only a few (20, corresponding to 29.4%) investigated the selected topic on humans. As expected, the condition most widely investigated was lung cancer (21/68), followed by pulmonary hypertension (7/68). All of the selected papers took into account the efficacy of the antagomiR treatment on the various diseases, whereas safety was rarely investigated (only in two cases out of 68). Overall, the use of antagomiR was seen to be efficient in downregulating the specific miRNA they are conceived for, with quite similar results either in vivo and in vitro and independently from the disease and the cell line they are used for. Overall, the usefulness of antagomiRs was demonstrated in humans, as well as in animals and cell lines without particular differences between those three aggregated groups. According to our results, the miRNA most frequently retrieved in lung conditions, therefore considered as “organ-specific,” although not “disease-specific,” appears to be miR-21, which has been found to be somewhat involved in lung conditions according to six of the selected papers [8,24,25,26,27,28]. However, miR-155 was also largely investigated and found to be involved in five works retrieved [29,30,31,32,72]. Further miRNAs frequently involved in lung conditions include miR-7 and miR-34, included in four articles, and miR-92 and miR-374, with three hits each. As evidenced, despite a handful miRNAs with multiple hits across the works included in our research, the only critical issue eventually identified for our approach concerns the right choice for the miRNA to be downregulated to achieve the expected result of the whole process. Indeed, even taking into account a single disease (e.g., lung cancer) on a similar population (e.g., on humans), a plethora of miRNAs with different functions can be identified as associated with the pathological process, therefore the selection of the right process to be blocked or, conversely, enhanced, is critical for good outcome of the process. Taking into account some specific disease categories, the first one to be analyzed, due to its numerical prevalence, was lung cancer. 

### 3.1. Lung Cancer

All the works retrieved showed an excellent efficacy of antagomiRs in blocking the action of the respective miRNAs both in vivo (on both humans and animals) and in vitro. The studies, taken singularly, have shown a wide heterogeneity of the mechanisms investigated and challenged with a respective wide difference in terms of miRNAs studied and antagomiRs employed. This applies both analyzing separately single sub-groups of samples and investigating the whole amount of studies together, making it difficult to identify a “principal” microRNA to be challenged in this specific domain.

#### 3.1.1. Humans

Eleven studies have specifically assessed the role of antagomiRs in human subjects with lung cancer, all of which demonstrating the usefulness of such compounds in blocking cancer-related cellular mechanisms, and even restraining the growth of tumors even in vivo (see, for example, He et al., 2019 [33] or Liu et al., 2011 [34]). The extreme heterogeneity of the miRNAs (and, consequently, antagomiRs) taken into account reveals the complexity of biological and cellular patterns of the lung cancer in vivo, suggesting the specific role of each compound for a given mechanism to be studied and tailored.

#### 3.1.2. Animals

Ten works investigating lung cancer in animals were retrieved, all of which used mice models for this assessment. AntagomiRs were demonstrated to be extremely efficient in modulating cancer growth, preventing it in most cases (see, for example, anti-miR135b, in Lin et al., 2013 [73]; anti-miR19a, in Liu et al., 2011 [34]; anti-miR494, in Mao et al., 2015 [74]), or even promoting tumor growth when experimentally needed (see anti-miR30c, in McCann et al., 2019 [75]).

#### 3.1.3. Cell lines

As expected, studies investigating lung cancer in cell lines are the vast majority with respect to the other sub-groups (20 studies were retrieved). Such studies confirmed in vitro the evidence arising in vivo, with antagomiRs able to modulate cancer growth depending on the outcome desired. Even on cell lines, the heterogeneity in terms of miRNAs investigated and antagomiRs employed was present, as already seen in humans and animals.

### 3.2. Bronchial Hypersensitivity

Under this category, studies dealing with asthma, COPD, allergic airway disease, airway hyper-responsiveness were included. Overall, eight studies were found, the majority of which investigating COPD (four studies) or asthma (three articles). Despite the heterogeneity in terms of miRNAs investigated, in this case promoted by both the relatively low number of studies retrieved and by the slightly different kind of diseases included in this category, miR-21 was included in several studies and its activity successfully challenged by its corresponding antagomiR. In summary, despite the differences in terms of category of samples studied and antagomiRs used, these compounds have demonstrated their usefulness also in this class of clinical conditions.

#### 3.2.1. Humans

Only four works investigated such diseases in humans, all of which dealing with COPD. Overall, the studies highlighted a positive contribution of antagomiRs in decreasing the clinical symptoms of the disease. In some cases, these molecules have even shown to improve clinical outcomes with the support of other compounds, like protein kinase R (see Hsu et al. 2016 [76]).

#### 3.2.2. Animals

Six studies were published on animal models, of which three investigated asthma, and one each COPD, airway disease, and airway hyper-responsiveness. All those studies took into account mice models, with miR-21 that was investigated in three out of the six works. Overall, the results were encouraging, with minor differences. Indeed, as observed by Collison et al. [24], just the inhibition of miR-145, but not miR-21 or let-7b, inhibited eosinophilic inflammation, mucus hypersecretion, TH2 cytokine production, and airway hyper-responsiveness, and also Plank et al. [30] on asthma observed that the use of the respective antagomiR (anti-miR-155-5p) reduced miR-155-5p expression, but failed to alter the disease phenotype.

#### 3.2.3. Cell lines

Just three studies, all concerning COPD, were published on cell lines. All the three took into account different miRNAs and corresponding antagomiRs, but the results were seen to be overall positive, except the above reported work by Hsu et al. [76], which highlighted the pivotal importance of combining antagomiRs with protein kinase R to increase antiviral stress granules formation, induction of p300 and IFN-β in COPD primary bronchial epithelial cells.

### 3.3. Pulmonary Hypertension

Six studies investigated pulmonary hypertension in the literature retrieved, mostly dealing with animal models. Overall, the various antagomiRs used (as previously, the heterogeneity in terms of miRNAs studied was high) demonstrated a good efficacy in challenging the corresponding miRNA and in improving the biological outcome.

#### 3.3.1. Humans

Only one work, published by Potus and colleagues [77] investigated the use of antagomiRs in pulmonary hypertension in humans, through biopsy specimens, with the surprising result that AntagomiR-126 in healthy CD31+ cells mimicked the PAH phenotype.

#### 3.3.2. Animals

Six works, including that by Potus and colleagues [77] above reported, have used animal models to this aim. All of them used either mice or rat models, with complete heterogeneity on the miRNAs studied. Overall, the treatment with antagomiRs improved the outcome with respect to pulmonary hypertension, except in the study by Gubrij et al. [78], where AntagomiR-223 was efficient in reducing levels of miR-223 in pulmonary artery and lungs of rats as compared to controls, but failed to attenuate pulmonary hypertension.

#### 3.3.3. Cell lines

Three articles also took into account cell lines on this specific topic. Despite using different compounds, tailoring different miRNAs, all the three works highlighted the positive role of antagomiRs in improving the outcome related to pulmonary hypertension, overall confirming the results obtained in vivo.

### 3.4. Lung Injury

In this category, acute lung injury and pulmonary inflammation were included. Overall, eight articles were included, four each for the two conditions above mentioned. No studies on human subjects were retrieved, while works dealing with animal models and cell lines were relatively numerous. As in other sub-groups and possibly due to the slight heterogeneity of the clinical conditions included in this category, just one miRNA was studied in more than one article even if taken into account in two studies by the same research team (miR-374a, investigated in Adyshev et al., [80,81]). Summarizing the results, even in this group of conditions, antagomiRs were extremely useful in improving the outcome both in vivo and in vitro, with no report of lack of efficacy in any of the studies retrieved.

#### 3.4.1. Animals

Six studies were retrieved on animal models. As in most of the cases previously reported, all of them were performed on mice models. Three of them investigated miRNAs in lung injury, two of them in lung inflammation and one [79] in both.

As stated, all the antagomiRs employed successfully reduced the pathological changes, starting from cellular level, in vivo, without evidence of inefficacy on the samples studied.

#### 3.4.2. Cell lines

Six studies were found also on cell lines. Three of them took into account lung inflammation, two lung injury and the one by Xie and colleagues [77] reported above both the conditions.

The results obtained confirmed the good reliability and efficacy of antagomiRs in improving the biomarkers of diseases in vitro, independently from the miRNA (and, therefore, the mechanism) tailored, with just one antagomiR, antagomiR-374a, that was used in more than one study [80,81], on lung injury and lung inflammation, respectively.

### 3.5. Other Conditions

In this category, several conditions, not matched by the groups above explained, were included, each of them featuring a small number of studies, and displaying positive results, overall.

#### 3.5.1. Humans

Four studies were performed on humans, one each about lung cell dysfunction, lung fibrosis, HIV infection and substance abuse, and tuberculosis. Given the different etiopathological processes of such conditions, also completely different mechanisms were investigated, and different miRNAs tailored. However, in all four cases, an improvement of the outcome was found in vivo, independently from the condition studied.

#### 3.5.2. Animals

Sixteen studies on animals were found for this macro-category, with the condition more largely studied being influenza (four studies). All but two of these studies were carried out on mice or rats, whereas Asquith et al. [85], studied chronic ethanol consumption in non-human primates, and Zhou et al. [88] assessed the effect of miRNAs and antagomiRs in influenza in one-month-old beagles.

Three studies [31,72,85] on chronic ethanol consumption, pneumonia, and systemic lupus erythematosus, investigated the role of miR-155, two [88,89] respectively on tuberculosis and influenza, of miR-143 and the others showed complete heterogeneity in the miRNAs studied.

Overall, antagomiRs demonstrated their efficacy in tailoring such conditions in vivo also on animal models, as already seen on humans, despite a lack of complete efficacy noticed by Chiba et al. [83] for miR-133b and let-7a antagomiRs in abnormal bronchial smooth muscle contraction studied in BALB/c mice.

#### 3.5.3. Cell Lines

Nineteen studies were included in this category, most of which were already mentioned in the two previous (humans and animals) groups, as performed both in vivo and in vitro. Three of them were performed on influenza [86,88,90] and three others on lung fibrosis [26,82,84]. Other conditions were more rarely studied.

Concerning the miRNAs studied, as above, a slight prevalence (three studies: Bhattacharyya et al. [29] on cystic fibrosis, Podsiad et al. [31] on pneumonia, Zhou et al. [86] on systemic lupus erythematosus) included miR-155, with the positive results about the use of the respective antagomiR seen in vivo that were confirmed in vitro. miR-143 was successfully tailored in two studies (Tamgue et al. [89] on tuberculosis, Zhou et al., [88] on influenza) confirming the positive retrievals already seen in vivo.

Overall, the other antagomiRs were also seen to be efficient in positively tailoring the activity of the respective miRNAs, thus improving the biological outcome of the various conditions they were applied to, also in vitro, despite minor evidence of lack of (or reduced) efficacy seen in Chiba et al. [83] on abnormal bronchial smooth muscle contraction and in Morales et al. [87] on SARS-CoV.

## 4. Discussion

It is well established that miRNAs are pleiotropic molecules involved in almost all major biological processes. This concept was particularly studied in the lung, where specific miRNAs demonstrated to cooperate with organ development and pulmonary diseases. During the last years, much data has been collected on this topic, with special regards to obstructive and restrictive lung diseases [91,92,93]. In fact, as Sessa and Hata [94] reported, a typical miRNAs expression profile was noticed and different miRNAs play an active role among different processes including hemostasis, viral infection, and inflammation. Lung-specific miRNAs can be used as novel biomarkers in lung disorders. To date, several pieces of research focused on specific lung disease miRNAs expression patterns. However, specific miRNAs expression profiles may be noticed also among different organs. Previously, Wang et al. [20] conducted a study on rats, demonstrating that two specific miRNAs (miR-195 and miR-200c) were peculiarly expressed in the lung, while eight miRNAs were co-expressed in the lung and heart and one miRNA was co-expressed in the lung and kidney. As interest increased on this topic, accessible databases as, for example, MiRmine were created in order to allow researchers to retrieve expression profiles of single or multiple miRNAs for a specific tissue or cell line, either normal or with disease information [95]. According to our results, miR-21 seems to be the most represented miRNA among lung conditions. MiR-21 is often up-regulated in lung carcinoma. This fact is believed to be a result of the capacity of miR-21 to inhibit tumor suppressor phosphatase and tensin-homolog [96]. Collison et al. [24] characterized miRNAs expression among house dust mite allergic mice. A group was treated with antagomiRs that inhibited the function of specific miRNAs in the lung, and the other group received standard steroid therapy with dexamethasone. Finally, inflammatory lesions and airway hyper-responsiveness were measured. Researchers found that, although miR-21 and let-7b were highly expressed during allergic inflammation, blockade of their function was ineffective at modulating the expression of disease. On the other hand, Kim et al. [27] conducted a study on BALB/c mice noticing that antagomiR-21 increased phosphatase and tensin homolog (PTEN) levels (*p* < 0.05). Treatment with Ant-21 reduced phosphoinositide 3-kinase (PI3K) activity and restored histone deacetylase (HDAC2) levels (*p* < 0.05), leading to suppression of airway hyper-responsiveness and to restore of steroid sensitivity to allergic airway disease. Lee et al. [60] also investigated allergic inflammation among mouse models, reporting that MiR-21 expression was down-regulated in mice lungs treated with anti-miR-21. In fact, specific antagomiR reduced both eosinophil count (*p* < 0.01) and Th2 cytokines levels, including IL-5 and IL-13 in mice BAL fluid (*p* < 0.05). MiRNA21 demonstrated positive effects also upon lung ischemia. Ischemia/reperfusion injury (IRI) is the primary cause of acute lung injury (ALI) and primary graft dysfunction (PGD) after lung transplantation [97]. Li et al. [28] conducted a study on murine lung ischemia/reperfusion (I/R) and in vitro hypoxia/reoxygenation (H/R) models demonstrating that pre-treatment of mesenchymal stromal cells with miR-21-5p antagomiR ameliorated IRI in the lung. Regarding PAH, Pullamsetti et al. [25] conducted a study both on animal models and cell lines demonstrating that Ant-17 and Ant-21 reduced right ventricular systolic pressure and pulmonary arterial muscularization. Moreover, Ant-17 decreased hypoxia-induced right ventricular hypertrophy and improved pulmonary artery acceleration time. In mice, Ant-17 therapy reduced right ventricular systolic pressure and total pulmonary vascular resistance index, stabilized cardiac output and reduced pulmonary vascular remodeling. In human pulmonary artery smooth muscle cells, Ant-17 increased the cyclin-dependent kinase inhibitor 1A (p21). MiRNA 21 demonstrated to have a role also upon lung fibrosis. In fact, Shentu et al. [26] demonstrated that human mesenchymal stem cell-derived extracellular vesicles (mEVs) do contain several specific miRNAs including 21-5p and 630. MEVs suppress TGFβ1-induced myofibroblastic differentiation of normal and idiopathic pulmonary fibrosis (IPF) lung fibroblasts, thus mitigating tissue fibrotic response. Investigating the role of miRNA regarding the pathogenesis and progression of lung fibrosis, Liu et al. [98] found that miR-21 was up-regulated both in the lungs of mice presenting with bleomycin-induced lung fibrosis and IPF patients. In this setting, miR-21 was highly expressed by myofibroblasts in the fibrotic lungs. Furthermore, researchers noticed that miR-21 reduced bleomycin-induced lung fibrosis in rats’ lungs.

**Figure 2:**
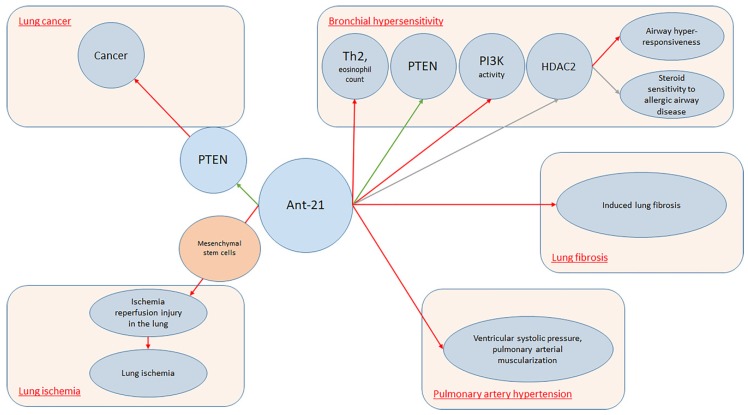
AntagomiR-21 in lung conditions.

Overall, a simple explanation of the mechanisms involving antagomiR-21 in lung conditions is provided in Figure 2.

Another miRNA molecule, which is widely expressed in several lung conditions, is miR-155. Several studies demonstrated that this molecule is upregulated in activated immune cells, such as T and B lymphocytes, macrophages, and dendritic cells (DCs). Indeed, miR-155 levels increase in response to inflammatory mediators. Moreover, miRNA-155 can regulate B-cell proliferation, malignancy, antibody production, and the differentiation and function of IL-17-producing helper T cells. Furthermore, miR-155 is induced by LPS, as well as other TLR ligands and proinflammatory cytokines [99,100,101]. As miR-155 is involved in several processes, it is feasible to understand its role upon many lung disorders. Basing on the hypothesis that miR-155 upregulation could inhibit IL-17 expression and therefore increase susceptibility to secondary bacterial pneumonia, Podsiad et [31] al. conducted a study on wild-type C57BL/6 mice and human lung macrophages in order to investigate the role of miR-155 and the respective antagoMiR upon viral and bacterial pneumonia. They concluded that miR-155 antagomiR ameliorated lung bacterial clearance compared with controls. MiR-155 plays a crucial role also upon ARDS. Triggering receptors expressed on myeloid cells (TREM) proteins are a family of immunoglobulin cell surface receptors expressed on myeloid cells and they are considered as amplifiers of Toll-like receptor (TLR)-induced inflammation. Experiments with antagomiR-155 confirmed that TREM-1-mediated changes were dependent on miR-155. Yuan et al. [32] conducted a study on wild-type C57BL/6J mice and bone marrow-derived macrophages demonstrating that TREM-1 boosted inflammatory response by inducing the expression of miR-155 in macrophages. Therefore, researchers inhibited TREM-1 using a nanomicellar strategy. Neutrophilic inflammation was reduced, thus suggesting that TREM-1 inhibition is a potential therapeutic target for neutrophilic lung inflammation and ARDS. Systemic lupus erythematosus (SLE) is a complex auto-immune disease which can involve several systems, including lungs. Diffuse alveolar hemorrhage (DAH) is a rare but severe complication of SLE and miR-155 showed to have a relevant role. In fact, Zhou et al. [86] found that miR-155 expression was up-regulated during the development of DAH, noticing that this molecule targets several pro-inflammatory mediators. The extent of lung inflammation was markedly reduced in miR-155–knockout (miR-1552/2) mice. Moreover, in vivo silencing of miR-155 using miR-155 antagomiR reduced the incidence of iatrogenic-induced DAH. MiR-155 cooperates with Th2 responses too. In fact, it is extensively expressed in the Th cell, DCs, and macrophages in the lung. MiR-155 was also found to be up-regulated in the nasal mucosa and airway smooth muscle cells of allergic asthmatic patients [27,100,101]. Recently, Plank et al. [30] conducted a study on murine asthmatic models noticing that MiR-155-5p is highly upregulated in mice. However, while targeting of miR-155-5p with a specific antagomiR resulted in specific inhibition in vivo, it was not able to alter the disease phenotype. Authors hypothesized that this could be due to the variation in antagomiR uptake, which demonstrated to be effective in myeloid cells and weak in lymphocytes. Cystic fibrosis (CF) is an autosomal recessive disease, due to the occurrence of cystic fibrosis transmembrane conductance regulator (CFTR) gene mutations and it is characterized by a variable cytokines pro-inflammatory milieu. Bhattacharyya et al. [29] demonstrated that antagomiR-155 down-regulates miR-155 expression suppressing IL-8 and other proinflammatory genes in CF cells.

**Figure 3:**
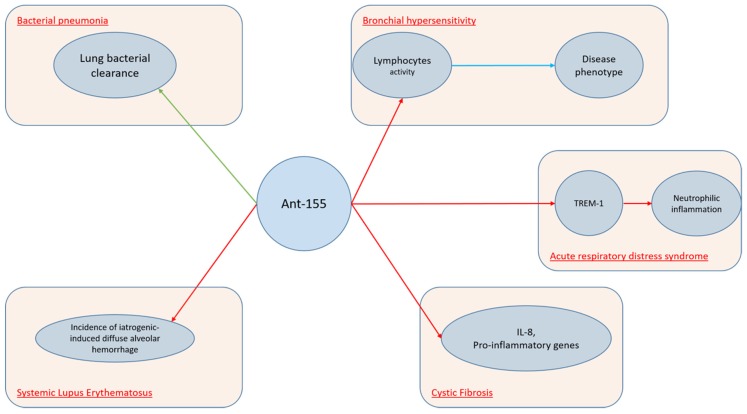
AntagomiR-155 in lung conditions.

The mechanisms involving antagomiR-155 in lung conditions are displayed in Figure 3.

## 5. Conclusions

This systematic review provides numerous shreds of evidence regarding dysregulation in miRNAs expression in lung diseases. It remains to understand the sources of the various miRNAs, and whether they have mainly disease- or organ-specific effects. However, these findings may contribute to a better definition of the complex network of miRNAs involved in lung diseases. Thus, miRNAs have been proposed as diagnostic or prognostic biomarkers and therapeutic targets for future treatments. Notably, antagomiRs are chemically modified oligonucleotides that are used to silence microRNAs, having the property of bind specifically to particular microRNAs. These could represent a therapeutic opportunity to modulate miRNA-induced, post-transcriptional mRNA regulation. To our best knowledge, this is the first article describing evidence on the involvement of miRNAs and the efficacy of respective antagomiRs in different lung diseases, including studies conducted on humans, animals, and cells. In conclusion, these findings provide new knowledge on the network of miRNAs in lung diseases and suggest that antagomiRs may represent a target for a specific therapy for these diseases.

**Key Points**

- Strong evidence confirmed that miRNAs play a crucial role in several pathologic mechanisms, therefore they have been proposed as diagnostic or prognostic biomarkers and therapeutic targets for future treatments.
- AntagomiRs are chemically modified oligonucleotides able of silencing microRNAs and are now emerging as novel therapeutic agents in several conditions.
- It has been widely demonstrated that miRNAs have a fundamental role among several lung conditions, moreover, some of these molecules proved to have a lung-specific tropism, thus suggesting the idea of lung-specific miRNAs patterns.
- To our best knowledge, this is the first article describing the evidence on the involvement of miRNAs and the efficacy of respective antagomiRs in different lung diseases, including studies conducted on humans, animals, and cells.
- Coherence between these groups has been demonstrated, thus suggesting the importance of developing new studies on these agents as target therapies.

## Figures and Tables

**Table 1:** Studies dealing with lung cancer (CDCP1: CUB-domain-containing protein 1; EC: endothelial cells; HUVECs: human umbilical vein endothelial cells; NOD-SCID: non-obese diabetic severe combined immune deficient; NSCLC: non-small cell lung cancer; SCID: severe combined immunodeficiency; TRAIL: tumor necrosis factor-related apoptosis-inducing ligand).

Lung Cancer				
**Humans**				
**Study**	**Disease**	**Sample**	**miRNAs**	**Findings**
Incoronato et al. (2010) [48]	Lung cancer	Cells: Calu-1 NSCLC cells; H460 cells Human Tissues: 18 snap-frozen normal and malignant lung tissues	miR-212	AntagomiR 212 increases PED protein expression and resistance to TRAIL treatment (*p* < 0.05)
Liu et al. (2011) [34]	Lung cancer	Cells: esophageal cancer cell lines KYSE150, KYSE410, KYSE70, EC109, and EC9706. Clinical samples: 28 ESCC and 28 normal tissue samples from surgically resected esophageal carcinoma specimens. Animals: five nude mice, injected with EC9706/miR-17-92 and EC9706/Control cells	miR-19a	Antagomir-19a treatment impairs tumor growth in vivo (*p* < 0.05)
Liang et al. (2015) [10]	Lung cancer	20 NSCLC patients, 20 controls	miR-223	miR-223 antagomir decreases tumor cell invasion and increases EPB41L3 in A549 cells (*p* < 0.001)
Wu et al. (2015) [64]	Lung cancer	Humans: 81 NSCLC patients (12 Stage I, 14 Stage II, 26 Stage III, 29 Stage IV), 41 controls. Animals: 4-week-old BALB/c nude mice. Cells: NSCLC cell lines (A549, H1299, H1975, Hcc827), human embryonic kidney (HEK) 293T cells	miR-25	miR-25 antagomir inhibited lung cancer growth via upregulation of MOAP1 in a mouse xenograft model (*p* < 0.01)
Sun et al. (2016) [62]	Lung cancer	Humans: NSCLC tumor tissue samples. Cells: human NSCLC cell lines A549, H1299, SPC-A-1, 95D, SK-MES-1, NCI-H520, NCI-H460, human normal lung epithelial cell line 16HBE	miR-346	AntagomiR-346 inhibited NSCLC cell growth and metastasis
Vera et al. (2017) [63]	Lung cancer	Fifteen human cancer cell lines, ovarian cancer samples (*n* = 138), high-grade serous carcinoma (*n* = 22); normal ovarian samples (*n* = 10), peripheral blood mononuclear cells (*n* = 10)	miR-7, miR-132, miR-335, and miR-148a	Relative miR-7 and MAFG expression levels decreased when treated with antagomir
Wu et al. (2016a) [65]	Lung cancer	Humans: patients with lung adenocarcinoma (*n* = 129) and lung squamous cell carcinoma (*n* = 54). Cells: human NSCLC cell line A549	miR-144-3p	miR-144-3p antagomir could enhance proliferation of IL-1β (*p* < 0.001)
Wu et al. (2016b) [66]	Lung cancer	Humans: human NSCLC tumor, adjacent normal lung tissues (*n* = 5). Cells: human NSCLC cell lines H358 and H23	miR-96	Antagomir-96 increased SAMD9 expression and the cisplatin-induced apoptosis and decreased cisplatin IC_50_ (*p* < 0.05)
Xie et al. (2017) [70]	Lung cancer	Humans: *n* = 83 patients with NSCLC. Animals: NSCLC xenograft nude mouse model. Cells: bronchoepithelial cell line BEAS-2B, A549, and HCC4006 human NSCLC cell line	miR-768-3p	miR-768-3p antagomir induced apoptosis and Fas/FasL expressional alteration of NSCLC cells; antimiR-768-3p transduction decreased viability, migration, invasion, MMP-2, MMP-9 activities in A549 and HCC4006 cells; antimiR-768-3p transfection inhibited growth and proliferation of NSCLC xenografts in nude mice (*p* < 0.05)
Zhu et al. (2018) [55]	Lung cancer	Humans: whole blood samples from patients with NSCLC and controls; tumor and nontumorous tissues obtained from NSCLC patients (*n* = 20). Cells: NSCLC cell lines SPCA1, A549, H2170	miR-92a	Proliferation of SPCA1, A549, H2170 inhibited by antimiR-92a (*p* < 0.05)
He et al. (2019) [33]	Lung adenocarcinoma	Human lung cancer cells A549, human bronchial epithelial cells BEAS-2B, human lung cancer cells SPCA1, SPC-A-1-BM human lung adenocarcinoma cell line	miR-499a-5p	Inhibition of miR-499a-5p by antagomirs restrained tumor growth in vivo (*p* < 0.01)
**Animals**				
**Study**	**Disease**	**Sample**	**miRNAs**	**Findings**
Cha et al. (2010) [40]	Lung cancer	Animals: *n* = 20 5-week-old male BALB/c nude mice. Cell lines: CL1-0, CL1-5 lung adenocarcinoma cell lines. H1299, PC14, H928, A549 lung cancer, MCF-7, MDA-MB231, MDA-MD431, T47D, SKBR3 breast cancer cell lines	miR-519c	Antagomir inhibition of miR-519c increased HIF-1α protein and enhanced angiogenic activity (*p* < 0.05)
Liu et al. (2011) [34]	Lung cancer	Cells: esophageal cancer cell lines KYSE150, KYSE410, KYSE70, EC109, EC9706. Clinical samples: 28 ESCC, 28 normal tissue samples from surgically resected esophageal carcinoma specimens. Animals: five nude mice injected with EC9706/miR-17-92 and EC9706/Control cells	miR-19a	Antagomir-19a treatment impairs tumor growth in vivo (*p* < 0.05)
Lin et al. (2013) [73]	Lung cancer	Animals: *n* = 10 mice. Cells: lung cancer cell lines CL1-0, CL1-1, CL1-5 and CL1-5-F4. A549, HOP-62, H441, CL141 cells, melanoma cell line UACC-257. H1299 and HEK-293 cells	miR-135b	miR-135b antagomirs suppress cancer cell invasion, orthotopic lung tumor growth and metastasis in mouse model (*p* < 0.05)
Shi et al. (2014) [59]	Lung cancer	Animals: immune-deficient NOD-SCID mice. Cells: human NSCLC cell lines A549, H460, and H1299	miR-34a	Expression of antimiR-34a in the CD44^lo^ H460 cells promoted tumor development (*p* < 0.05)
Chiu et al. (2015) [42]	Lung cancer	Human cells: human lung cancer cell lines CL1-0, F4, Bm7, Bm7brmx2, A549, H1299. Animals: Lung cancer cells injected into SCID mice	miR-218	CDCP1 protein levels increased in cells treated with miR-218 antagomirs (*p* < 0.05)
Mao et al. (2015) [74]	Lung cancer	Human cells: HUVECs, tumor cell lines A549, H1299, HCC827. Animals: male BALB/c nude mice	miR-494	MiR-494 antagomiR inhibited angiogenesis and attenuated the growth of tumor xenografts in nude mice (*p* < 0.05)
Wu et al. (2015) [64]	Lung cancer	Humans: 81 NSCLC patients (12 Stage I, 14 Stage II, 26 Stage III, 29 Stage IV), 41 controls. Animals: BALB/c nude mice. Cells: NSCLC cell lines A549, H1299, H1975, Hcc827, HEK 293T cells	miR-25	miR-25 antagomir inhibited lung cancer growth via upregulation of MOAP1 in a mouse xenograft model (*p* < 0.01)
Xie et al. (2017) [70]	Lung cancer	Humans: *n* = 83 NSCLC patients. Animals: NSCLC xenograft nude mouse model. Cells: bronchoepithelial cell line BEAS-2B, A549 and HCC4006 human NSCLC cell line	miR-768-3p	miR-768-3p antagomir induced distinctly apoptosis and Fas/FasL expressional alteration of NSCLC cells; miR-768-3p antagomir transduction decreased viability, migration, invasion, MMP-2, and MMP-9 activities in A549 and HCC4006 cells; miR-768-3p antagomir transfection inhibited the growth and proliferation of NSCLC xenografts in nude mice (*p* < 0.05)
Zhang et al. (2018) [53]	Lung cancer	Animals: 20 male BALB/c nu/nu mice. Cells: EGFR mutant non-small cell lung cancer cell line PC-9	miR-214	AntagomiR-214 reversed gefitinib resistance conferred by PC-9GR-derived exosomes in vitro and reversed gefitinib resistance in vivo (*p* < 0.01 in both cases)
McCann et al. (2019) [75]	Lung cancer	Animals: mice models. Cells: primary endothelial cells isolated from normal or tumor tissue from mice	miR-30c	miR-30c antagomiRs promoted PAI-1–dependent tumor growth and increased fibrin abundance (*p* < 0.05)
**Cell Lines**				
**Study**	**Disease**	**Sample**	**miRNAs**	**Findings**
Cha et al. (2010) [40]	Lung cancer	Animals: *n* = 20 5-week-old male BALB/c nude mice. Cell lines: CL1-0, CL1-5 lung adenocarcinoma cell lines. H1299, PC14, H928, A549 lung cancer, MCF-7, MDA-MB231, MDA-MD431, T47D, SKBR3 breast cancer cell lines	miR-519c	Antagomir inhibition of miR-519c increased the level of HIF-1α protein and enhanced angiogenic activity (*p* < 0.05)
Guo et al. (2010) [46]	Lung cancer	Human small lung cancer cell line NCI-H69 and drug-resistant subline H69AR	miR-134, miR-379, miR-495	Sensitivity to anti-cancer drugs Cisplatin, Etoposide, and Doxorubicin reduced after transfection of drug-resistant H69AR cells with the antagomirs of miR-134, miR-379 and miR-495 (*p* < 0.05)
Incoronato et al. (2010) [48]	Lung cancer	Cells: Calu-1 NSCLC cells; H460 cells Human Tissues: 18 snap-frozen normal and malignant lung tissues	miR-212	AntagomiR 212 increases PED protein expression and resistance to TRAIL treatment (*p* < 0.05)
Liu et al. (2011) [34]	Lung cancer	Cells: esophageal cancer cell lines KYSE150, KYSE410, KYSE70, EC109, and EC9706. Clinical samples: 28 ESCC and 28 normal tissue samples from surgically resected esophageal carcinoma specimens. Animals: five nude mice, injected with EC9706/miR-17-92 and EC9706/Control cells	miR-19a	Antagomir-19a treatment impairs tumor growth in vivo (*p* < 0.05)
Lin et al. (2013) [73]	Lung cancer	Animals: *n* = 10 mice. Cells: lung cancer cell lines CL1-0, CL1-1, CL1-5, and CL1-5-F4. A549, HOP-62, H441, CL141 cells, melanoma cell line UACC-257. H1299 and HEK-293 cells	miR-135b	miR-135b antagomirs suppress cancer cell invasion, orthotopic lung tumor growth, and metastasis in mouse model (*p* < 0.05)
Shi et al. (2014) [59]	Lung cancer	Animals: Immune-deficient NOD-SCID mice. Cells: human NSCLC cell lines A549, H460, and H1299	miR-34a	Expression of miR-34a antagomirs in the CD44^lo^ H460 cells promoted tumor development (*p* < 0.05)
Silveyra et al. (2014) [60]	Lung cancer	Cells: lung adenocarcinoma cell line NCI- H441, three Chinese hamster ovary (CHOK1) cell lines expressing the human SP-A variants 1A^0^, 6A^2^, and 6A^4^	miR-183, miR-4507	antagomir-183 reversed the effects of mir-183 on SP-A mRNA levels (*p* < 0.05)
Chiu et al. (2015) [42]	Lung cancer	Human cells: human lung cancer cell lines CL1-0, F4, Bm7, Bm7brmx2, A549, and H1299. Animals: lung cancer cells injected intracardially into SCID mice	miR-218	CDCP1 levels increased in cells treated with antimiR-218 (*p* < 0.05)
Mao et al. (2015) [74]	Lung cancer	Human cells: HUVECs, tumor cell lines A549, H1299, HCC827. Animals: male BALB/c nude mice	miR-494	AntimiR-494 inhibited angiogenesis and attenuated the growth of tumor xenografts in nude mice (*p* < 0.05)
Sun et al. (2015) [61]	Lung cancer	NSCLC cell line A549	miR-1290	AntimiR-1290 suppressed tumor volume and weight initiated by CD133^+^ cells in vivo; Anti-miR-1290 inhibited proliferation, clonogenicity, invasion, and migration of CD133^+^ (*p* < 0.05)
Wu et al. (2015) [64]	Lung cancer	Humans: 81 NSCLC patients (12 Stage I, 14 Stage II, 26 Stage III, 29 Stage IV), 41 controls. Animals: BALB/c nude mice. Cells: NSCLC cell lines A549, H1299, H1975, Hcc827, HEK 293T cells	miR-25	miR-25 antagomir inhibited lung cancer growth via upregulation of MOAP1 in mice (*p* < 0.01)
Sun et al. (2016) [62]	Lung cancer	Humans: NSCLC tumor tissues. Cells: human NSCLC cell lines A549, H1299, SPC-A-1, 95D, SK-MES-1, NCI-H520, NCI-H460, human normal lung epithelial cell line 16HBE	miR-346	AntimiR-346 inhibited NSCLC cell growth and metastasis
Wu et al. (2016a) [65]	Lung cancer	Humans: patients with lung adenocarcinoma (*n* = 129) and lung squamous cell carcinoma (*n* = 54). Cells: human NSCLC cell line A549	miR-144-3p	miR-144-3p antagomir could enhance IL-1β proliferation (*p* < 0.001)
Wu et al. (2016b) [66]	Lung cancer	Humans: human NSCLC tumor, adjacent normal lung tissues (*n* = 5). Cells: human NSCLC cell lines H358 and H23	miR-96	Antagomir-96 increased SAMD9 expression and the cisplatin-induced apoptosis, it decreased cisplatin IC_50_ (*p* < 0.05)
Vera et al. (2017) [63]	Lung cancer	15 human cancer cell lines, ovarian cancer samples (*n* = 138), high-grade serous carcinoma (*n* = 22); normal ovarian samples (*n* = 10), peripheral blood mononuclear cells (*n* = 10)	miR-7, miR-132, miR-335, and miR-148a	Relative miR-7 and MAFG expression levels decreased when treated with antagomir
Xie et al. (2017) [70]	Lung cancer	Humans: *n* = 83 NSCLC patients. Animals: NSCLC xenograft nude mouse model. Cells: bronchoepithelial cell line BEAS-2B, A549 and HCC4006 human NSCLC cell line	miR-768-3p	miR-768-3p antagomir induced distinctly apoptosis and Fas/FasL expressional alteration of NSCLC cells; miR-768-3p antagomir transduction decreased viability, migration, invasion, MMP-2, and MMP-9 activities in A549 and HCC4006 cells; miR-768-3p antagomir transfection inhibited the growth and proliferation of NSCLC xenografts in nude mice (*p* < 0.05)
Zhang et al. (2018) [53]	Lung cancer	Animals: 20 male BALB/c nu/nu mice. Cells: EGFR mutant non-small cell lung cancer cell line PC-9	miR-214	AntagomiR-214 reversed gefitinib resistance in vitro and in vivo (*p* < 0.01)
Zhu et al. (2018) [55]	Lung cancer	Humans: whole blood samples collected from healthy individuals and NSCLC patients; tumor and adjacent nontumorous tissues from NSCLC patients (*n* = 20). Cells: NSCLC cell lines SPCA1, A549, H2170	miR-92a	Proliferation of SPCA1, A549, H2170 inhibited by antimiR-92a (*p* < 0.05)
He et al. (2019) [33]	Lung adenocarcinoma	Human lung cancer cells A549, human bronchial epithelial cells BEAS-2B, human lung cancer cells SPCA1, SPC-A-1-BM	miR-499a-5p	Inhibition of miR-499a-5p by antagomirs restrained tumor growth in vivo (*p* < 0.01)
McCann et al. (2019) [75]	Lung cancer	Animals: mice models. Cells: primary endothelial cells isolated from normal or tumor tissue from mice	miR-30c	miR-30c antagomiRs promoted PAI-1–dependent tumor growth and increased fibrin abundance (*p* < 0.05)

**Table 2:** Studies dealing with bronchial hypersensitivity (avSG: antiviral stress granules; COPD: chronic obstructive pulmonary disease; pBECs: primary bronchial epithelial cells; PH: pulmonary hypertension; PKR: protein kinase R; PTEN: phosphatase and tensin homolog; SAECs: small airway epithelial cells).

Bronchial Hypersensitivity				
**Humans**				
**Study**	**Disease**	**Sample**	**miRNAs**	**Findings**
Baker et al. (2016) [37]	COPD	Peripheral lung samples from COPD patients and controls; airway epithelial cells	miR-34a	miR-34a antagomirs increased SIRT1 (*p* < 0.01)/-6 (*p* < 0.05) mRNA levels, decreasing cellular senescence markers in COPD (*p* < 0.05)
Hsu et al. (2016) [76]	COPD	Five COPD, five smokers, five controls	miR-132	Ectopic expression of PKR or miR-132 antagomiR alone failed to restore IFN-β induction (*p* > 0.05), co-treatment increased avSG formation, induction of p300, and IFN-β in COPD pBECs (*p* < 0.05)
Jiang et al. (2018) [49]	COPD	Humans: 73 patients with PH, 32 controls. Animals: hypoxia-induced PH mice	miR-190a-5p	Antagomir-190a-5p reduced right ventricular systolic pressure (*p* < 0.01) and enhanced KLF15 expression (*p* < 0.0001) in lung tissue
Baker et al. (2019) [38]	COPD	30 COPD/18 controls: lung tissue from tissue bank; 14 COPD, 10 non-smoking controls: human primary SAECs cultured; 13 COPD, five controls: sputum samples collected	miR-570-3p	Inhibition of elevated miR-570-3p in COPD small airway epithelial cells, using an antagomir, restores sirtuin-1, and suppresses markers of cellular senescence, restoring cellular growth (*p* < 0.05)
**Animals**				
**Study**	**Disease**	**Sample**	**miRNAs**	**Findings**
Collison et al. (2011) [24]	Allergic airway disease	BALB/c mice sensitized with house dust mite	miR-145, miR-21, let-7b	Inhibition of miR-145 (*p* < 0.05), but not miR-21 or let-7b (both *p* > 0.05), inhibited eosinophilic inflammation, mucus hypersecretion, TH2 cytokine production, and airway hyper-responsiveness
Li et al. (2015) [50]	Airway hyper-responsiveness	Wild-type specific pathogen-free BALB/c mice	miR-9	AntagomiR-9 increased PP2A activity and GR nuclear translocation in macrophages (*p* < 0.05), restored steroid sensitivity in steroid-resistant airway hyper-responsiveness
Plank et al. (2015) [30]	Asthma	Specific pathogen-free BALB/c mice	miR-155-5p	Antagomir administration reduced miR-155-5p expression (*p* < 0.01), but failed to alter the disease phenotype (*p* > 0.05). It exhibits poor uptake in lymphocytes
Kim et al. (2017) [27]	Asthma	BALB/c mice	miR-21	Antagomir-21 increased PTEN levels (*p* < 0.05). Treatment with Ant-21 reduced PI3K activity and restored HDAC2 levels (*p* < 0.05), suppressing airway hyper-responsiveness and restoring steroid sensitivity to allergic airway disease
Lee et al. (2017) [35]	Acute bronchial asthma	BALB/c mice sensitized and challenged with ovalbumin	miR-21	MiR-21 expression down-regulated in mice lungs treated with anti-miR-21. It reduced total cell (*p* < 0.001) and eosinophil counts (*p* < 0.01) in BAL fluid and the levels of IL-5 and IL-13 (*p* < 0.05)
Jiang et al. (2018) [49]	COPD	Humans: 73 patients with PH, 32 controls. Animals: hypoxia-induced PH mice	miR-190a-5p	Antagomir-190a-5p reduced right ventricular systolic pressure (*p* < 0.01) and enhanced the KLF15 expression levels (*p* < 0.0001) in lung tissue
**Cell Lines**				
**Study**	**Disease**	**Sample**	**miRNAs**	**Findings**
Baker et al. (2016) [37]	COPD	Peripheral lung samples from COPD patients and controls; airway epithelial cells	miR-34a	miR-34a antagomirs increased SIRT1 (*p* < 0.01)/-6 (*p* < 0.05) mRNA levels, decreasing markers of cellular senescence in airway epithelial cells from COPD (*p* < 0.05)
Hsu et al. (2016) [76]	COPD	Five COPD, five smokers, five controls	miR-132	Ectopic expression of PKR or miR-132 antagomiR alone failed to restore IFN-β induction (*p* > 0.05), co-treatment increased avSG formation, induction of p300 and IFN-β in COPD pBECs (*p* < 0.05)
Baker et al. (2019) [38]	COPD	30 COPD/18 controls: lung tissue from a tissue bank; 14 COPD, 10 non-smoking controls: human primary SAECs cultured; 13 COPD, five controls: sputum samples collected	miR-570-3p	Inhibition of elevated miR-570-3p in COPD small airway epithelial cells, using an antagomir, restores sirtuin-1 and suppresses markers of cellular senescence, restoring cellular growth (*p* < 0.05)

**Table 3:** Studies dealing with pulmonary hypertension (EC: endothelial cells; HPASMCs: human pulmonary arterial smooth muscle cells; MCT-PAH: monocrotaline pulmonary artery hypertension; PAH: pulmonary artery hypertension; SMC: smooth muscle cells).

Pulmonary Hypertension				
**Humans**				
**Study**	**Disease**	**Sample**	**miRNAs**	**Findings**
Potus et al. (2014) [77]	Pulmonary hypertension	Humans: percutaneous biopsy of vastus lateralis (*n* = 11 patients, *n* = 9 controls). Animals: male Sprague-Dawley rats. Cells: CD311 cells isolated from two idiopathic PAH, two heritable PAH, and three control quadriceps biopsies	miR-126	AntagomiR-126 in healthy CD31^+^ cells mimicked the PAH phenotype. In skeletal muscle of healthy rats, it decreased muscle capillarity (*p* < 0.05) and exercise tolerance in treadmill tests (*p* < 0.05)
**Animals**				
**Study**	**Disease**	**Sample**	**miRNAs**	**Findings**
Pullamsetti et al. (2012) [25]	Pulmonary hypertension	Animals: mice and rat models. Cells: pooled human umbilical vein ECs and human pulmonary artery SMCs	miR-17, miR-21, miR-92a	Ant-17 and Ant-21 reduced right ventricular systolic pressure, all antagomirs decreased pulmonary arterial muscularization. Ant-17 reduced hypoxia-induced right ventricular hypertrophy, improved pulmonary artery acceleration time. In rats, Ant-17 decreased right ventricular systolic pressure and total pulmonary vascular resistance index, increased pulmonary artery acceleration time, normalized cardiac output, and decreased pulmonary vascular remodeling. In human pulmonary artery smooth muscle cells, Ant-17 increased p21
Brock et al. (2014) [39]	Pulmonary hypertension	Animals: four mice samples (three in hypoxic condition, one control). In vitro: HPASMCs	miR-20a	Animals: AntagomiR-20a enhanced BMPR2 expression levels in lung tissues by 59.3% (*p* < 0.001), reduced wall thickness (*p* < 0.01), luminal occlusion of small pulmonary arteries (*p* < 0.001) and right ventricular hypertrophy (*p* < 0.01). In vitro: Transfection of HPASMCs with antimiR-20a activates downstream targets of BMPR2 increasing activation of Id-1 and Id-2 (*p* < 0.05). HPASMCs proliferation reduced upon transfection with antagomiR-20a (*p* < 0.05)
Potus et al. (2014) [77]	Pulmonary hypertension	Humans: percutaneous biopsy of vastus lateralis (*n* = 11 patients, *n* = 9 controls). Animals: male Sprague-Dawley rats. Cells: CD311 cells isolated from two idiopathic PAH, two heritable PAH, and three control quadriceps biopsies	miR-126	AntagomiR-126 in healthy CD31^+^ cells mimicked the PAH phenotype. In skeletal muscle of healthy rats, it decreased muscle capillarity (*p* < 0.05) and exercise tolerance in treadmill tests (*p* < 0.05)
Sharma et al. (2015) [57]	Pulmonary hypertension	Male Sprague-Dawley rats	miR-206	Knockdown of miR-206 reduced right ventricular pressure and right ventricular hypertrophy index
Gubrij et al. (2016) [78]	Pulmonary hypertension	MCT-PAH rats	miR-223	A223 reduced levels of miR-223 in pulmonary artery and lungs of MCT PAH rats as compared to controls (*p* < 0.05), but did not attenuate MCT PAH (*p* > 0.05)
Mondejar-Parreño et al. (2019) [51]	Pulmonary hypertension	Pathogen-free male Wistar rats	miR-1	AntagomiR-1 prevented (*p* < 0.05) hypoxia-induced decline in voltage-dependent potassium channel Kv1.5 currents
**Cell Lines**				
**Study**	**Disease**	**Sample**	**miRNAs**	**Findings**
Pullamsetti et al. (2012) [25]	Pulmonary hypertension	Animals: mice and rat models. Cells: pooled human umbilical vein ECs and human pulmonary artery SMCs	miR-17, miR-21, miR-92a	Ant-17 and Ant-21 reduced right ventricular systolic pressure, all antagomirs decreased pulmonary arterial muscularization. Ant-17 reduced hypoxia-induced right ventricular hypertrophy, improved pulmonary artery acceleration time. In rats, Ant-17 decreased right ventricular systolic pressure and total pulmonary vascular resistance index, increased pulmonary artery acceleration time, normalized cardiac output, and decreased pulmonary vascular remodeling. In human pulmonary artery smooth muscle cells, Ant-17 increased p21
Brock et al. (2014) [39]	Pulmonary hypertension	Animals: four mice samples (three in hypoxic condition, one control). In vitro: HPASMCs	miR-20a	Animals: AntagomiR-20a enhanced BMPR2 expression levels in lung tissues by 59.3% (*p* < 0.001), reduced wall thickness (*p* < 0.01), luminal occlusion of small pulmonary arteries (*p* < 0.001) and right ventricular hypertrophy (*p* < 0.01). In vitro: Transfection of HPASMCs with antimiR-20a activates downstream targets of BMPR2 increasing activation of Id-1 and Id-2 (*p* < 0.05). HPASMCs proliferation reduced upon transfection with antagomiR-20a (*p* < 0.05)
Potus et al. (2014) [77]	Pulmonary hypertension	Humans: percutaneous biopsy of vastus lateralis (*n* = 11 patients, *n* = 9 controls). Animals: male Sprague-Dawley rats. Cells: CD311 cells isolated from two idiopathic PAH, two heritable PAH, and three control quadriceps biopsies	miR-126	AntagomiR-126 in healthy CD31^+^ cells mimicked the PAH phenotype. In skeletal muscle of healthy rats, it decreased muscle capillarity (*p* < 0.05) and exercise tolerance in treadmill tests (*p* < 0.05)

**Table 4:** Studies dealing with lung injury (BALF: bronchoalveolar lavage fluid; BMDM: bone marrow-derived macrophages; PBEF: pre-B-cell colony-enhancing factor).

Lung Injury				
**Animals**				
**Study**	**Disease**	**Sample**	**miRNAs**	**Findings**
Xu et al. (2014) [68]	Lung injury	Animals: healthy male C57BL/6 mice. Cells: Epithelial cells	miR-17	miR-17 antagomir increased the expression of FoxA1 in Acute Lung Injury mice (*p* < 0.05)
Yuan et al. (2015) [32]	Lung inflammation	Animals: male wild-type C57BL/6J mice. Cells: bone marrow-derived macrophages	miR-155	Increased expression of miR155 by mTREM-1 suppressed by antimiR-155 (*p* < 0.05)
Fu et al. (2018) [45]	Pulmonary inflammation	Animals: male BALB/c mice. Cells: murine macrophage RAW264.7 cells	miR-92a	Antagomir-92a reduced pathological changes associated with lung inflammation, reduces lung wet/dry ratio (*p* < 0.01), and Evans blue dye extravasation (*p* < 0.01). Inhibition of miR-92a reduced the repression of TNF-α, IL-1β, IL-6 (*p* < 0.01) in lung tissues
Wu et al. (2018) [67]	Acute lung injury	MK2 deficient mice (C57BL/6) (B6.129P2-Mapkapk2^tm1Dgen/J^, and MK2^flox/flox^ mice	Let-7e	Transfection of anti-let-7e into MK2^-/-^ BMDM rescued LPS-induced expression of TNF-α, IL-6, and MIP-2 (*p* < 0.05)
Xie et al. (2018) [79]	Lung inflammation, lung injury	Animals: male C57BL/6 mice. Cells: RAW264.7 cells	miR-34b-5p	miR-34b-5p antagomir in vivo inhibited miR-34b-5p up-regulation, reduced inflammatory cytokine release, decreased alveolar epithelial cell apoptosis, attenuated lung inflammation, improved survival by targeting PGRN during acute lung injury (*p* < 0.05)
Huang et al. (2019) [47]	Acute Lung Injury	Sixty healthy male-specific pathogen free C57BL/6 mice	miR-27b	Downregulation of miR-27b decreased the levels of IL-1β, IL-6, and TNF-α in BALF of Acute Lung Injury mice (*p* < 0.05)
**Cell Lines**				
**Study**	**Disease**	**Sample**	**miRNAs**	**Findings**
Adyshev et al. (2013) [80]	Lung injury	Human pulmonary artery endothelial cells	hsa-miR-374a, hsa-miR-374b, hsa-miR-520c-3p, hsa-miR-1290	Antagomirs for each MYLK miRNA increased 3′UTR luciferase activity (1.2–2.3 FI) and rescued the decreased MLCK-3′UTR reporter activity produced by miRNA mimics (70%–110% increases for each miRNA; *p* < 0.05)
Adyshev et al. (2014) [81]	Lung inflammation	Human pulmonary artery endothelial cells	hsa-miR-374a, hsa-miR-568	Antagomirs for each PBEF/NAMPT miRNA increased the endogenous PBEF/NAMPTmRNA and protein levels and 3′-UTR luciferase activity compared with controls (*p* < 0.05)
Xu et al. (2014) [68]	Lung injury	Animals: healthy male C57BL/6 mice. Cells: epithelial cells	miR-17	miR-17 antagomir increased the expression of FoxA1 in Acute Lung Injury mice (*p* < 0.05)
Yuan et al. (2015) [32]	Lung inflammation	Animals: Male wild-type C57BL/6J mice. Cells: bone marrow-derived macrophages	miR-155	Increased expression of miR155 by mTREM-1 suppressed by antagomir against miR-155 (*p* < 0.05)
Fu et al. (2018) [45]	Pulmonary inflammation	Animals: male BALB/c mice. Cells: murine macrophage RAW264.7 cells	miR-92a	Antagomir-92a reduced pathological changes associated with lung inflammation, reduces lung wet/dry ratio (*p* < 0.01), and Evans blue dye extravasation (*p* < 0.01). Inhibition of miR-92a ameliorated the inflammatory response by reducing the repression of TNF-α, IL-1β, IL-6 (*p* < 0.01) in lung tissues
Xie et al. (2018) [79]	Lung inflammation, lung injury	Animals: male C57BL/6 mice. Cells: RAW264.7 cells	miR-34b-5p	miR-34b-5p antagomir in vivo inhibited miR-34b-5p up-regulation, reduced inflammatory cytokine release, decreased alveolar epithelial cell apoptosis, attenuated lung inflammation, improved survival by targeting PGRN during acute lung injury (*p* < 0.05)

**Table 5:** Studies dealing with other conditions (ACE2: Angiotensin Converting Enzyme 2; BMDM: bone marrow-derived macrophages; BSM: bronchial smooth muscle; CF: Cystic Fibrosis; CIV: canine influenza virus; hBSMCs: human BSM cells; HLMECs: human lung microvascular endothelial cells; HPASMCs: human pulmonary arterial smooth muscle cells; HS: honeysuckle; HUVECs: human umbilical vein endothelial cells; Igfbp5: insulin-like growth factor binding protein 5; MDCK: Madin-Darby Canine Kidney; MDM: monocyte-derived macrophages; MSC: mesenchymal stromal cell; PASMC: pulmonary arterial smooth muscle cells; PBMC: peripheral blood mononuclear cells; SARS-CoV: severe acute respiratory syndrome coronavirus).

Other Conditions				
**Humans**				
**Study**	**Disease**	**Sample**	**miRNAs**	**Findings**
Chatterjee et al. (2014) [41]	Lung cell dysfunction	HLMECs, HUVECs	miR-147b	MiR-147b antagomir increased total and cell surface expression of ADAM15 in endothelial cells (*p* < 0.05)
Ge et al. (2016) [82]	Lung fibrosis	Humans: bronchial epithelia from lung transplant patients. Cells: primary fibroblasts isolated from human lungs	miR-323a-3p	Antagomirs for miR-323a-3p augment murine lung fibrosis after bleomycin injury (*p* < 0.05)
Sharma et al. (2018) [58]	HIV infection and substance abuse	Human monocyte derived macrophages, HPASMCs	miR-130a	Transfection of HPASMCs with antagomir-130a–ameliorated the extracellular vesicles-induced effect (*p* < 0.001)
Yuan et al. (2018) [69]	Tuberculosis	Fifty patients, 20 controls. Monocytes isolated from peripheral blood mononuclear cells	miR-196b-5p	antagomir-196b-5p promoted Bacillus Calmette–Guérin uptake in MDMs or differentiated U937 cells (*p* < 0.05)
**Animals**				
**Study**	**Disease**	**Sample**	**miRNAs**	**Findings**
Krützfeldt et al. (2005) [7]	Various conditions	Mice models	miR-16, miR-122, miR-192, miR-194	Intravenous administration of antagomirs reduced miRNA levels in liver, lung, kidney, heart, intestine, fat, skin, bone marrow, muscle, ovaries, and adrenals
Chiba et al. (2009) [83]	Abnormal BSM contraction	BSM cells, bronchial tissues of BALB/c mice	miR-133a	Up-regulation of RhoA when endogenous miR-133a function inhibited by its antagomir in hBSMCs (*p* < 0.05). No effect (*p* > 0.05) of miR-133b and let-7a antagomirs
Pandit et al. (2010) [84]	Pulmonary Fibrosis	Animals: mice models. Cells: 10 Idiopathic Pulmonary Fibrosis, 10 control tissues	Let-7d	Let-7d antagomir decreased expression of CDH1 and TJP1 and increased COL1A1 and HMGA2 expression in the lungs (*p* < 0.05)
Rosenberger et al. (2012) [56]	Influenza	C57Bl/6, MyD88^null^ mice	miR-451	Three types of primary dendritic cells treated with antagomirs against miR-451 secreted elevated levels of IL-6 (p< 0.01), TNF (*p* < 0.05), CCL5/RANTES (*p* < 0.05), and CCL3/MIP1α (*p* < 0.01)
Asquith et al. (2014) [85]	Chronic ethanol consumption	From non-human primates: PBMC, mesenteric and tracheobronchial lymph nodes, jejunum, duodenum, ileum, and descending colon	miR-181a, miR-221 (in PBMC), miR-155 (in colon)	Transfection of miRNA antagomirs upregulated both STAT-3 (*p* < 0.05)/ARNT (*p* < 0.001), VEGF (*p* < 0.05)/HGF (*p* < 0.01)/G-CSF (*p* < 0.05)
Zhang et al. (2015) [52]	Various disorders	Animals: BALB/c mice. Cells: 4T1 murine breast cancer cells	miR-10b	Antagomir-10b and PTX delivered by D-Lip delays the growth of 4T1 tumors and reduce lung metastases; Hoxd10 expression in tumors up-regulated (*p* < 0.01)
Zhou et al. (2015) [54]	Influenza	Animals: six groups of mice (five mice per group), including control group. Cells: MDCK cells	miR-2911	Inhibitory effect of HS decoction on viral replication abolished by anti-miR2911 (*p* < 0.05)
Podsiad et al. (2015) [31]	Pneumonia	Animals: wild-type C57BL/6 mice. Cells: Human lung macrophages	miR-155	miR-155 antagomir improved lung bacterial clearance by 4.2-fold
Zhou et al. (2016) [86]	Systemic Lupus Erythematosus	Animals: C57BL/6J (B6) and B6.Cg-Mir155^tm1.1Rsky/J^ mice. Cells: Hepa 1-6 cells	miR-155	Disease progression reduced by 20% by in vivo using of antimiR-155
Ma et al. (2017) [71]	Hypoxia	Animals: adult male Wistar rats. Cells: PASMC cultured	miR-125a	miR-125a antagomir mimicked the hypoxic damage effects to mitochondrial homeostasis (*p* < 0.05)
Morales et al. (2017) [87]	SARS-CoV	Animals: female mice. Cells: mouse delayed brain tumor cells expressing the murine SARS-CoV receptor ACE2	svRNA-nsp3.1, svRNA-nsp3.2, svRNA-N, miR-877	Antagomirs reduced partially (svRNA-nsp3.1), or totally (svRNA-nsp3.2, svRNA-N, miR-877), the luciferase activity
Zhou et al. (2017) [88]	Influenza	Animals: beagles. Cells: MDCK cells	cfa-miR-143	Anti-cfa-miR-143 caused upregulation of Igfbp5 in CIV-infected MDCK cells
Fehl et al. (2019) [44]	Bronchopulmonary dysplasia	Newborn C57BL/6J mice	N/A	AntagomiRs impacted lung volume (*p* < 0.05), septal thickness (*p* < 0.01), and the transcriptome (*p* < 0.05) of developing mouse lungs
Li et al. (2019) [28]	Lung ischemia	Mail C57/BL6 mice	miR-21-5p	Pre-treatment of MSCs with miR-21-5p antagomir decreased miR-21-5p expression level in exosomes secreted
Tamgue et al. (2019) [89]	Tuberculosis	Bone marrow-derived macrophages generated from male BALB/c mice	miR-143, miR-365	Antagomirs for miR-143 and miR-365 decreased the intracellular growth of Mtb HN878, reduced the production of IL-6 (*p* < 0.001) and CCL5 (*p* < 0.01 for miR-143, *p* < 0.05 for miR-365) and promoted the apoptotic death of Mtb HN878-infected BMDMs (*p* < 0.01 for miR-143, *p* < 0.05 for miR-365)
Zhang et al. (2019) [90]	Influenza	Animals: C57BL/6 mice. Cells: human pulmonary epithelial cell line A549	miR-146a	Downregulation of miR-146a inhibits Influenza A Virus replication by enhancing type I IFN response through TRAF6 in vitro and in vivo (*p* < 0.01)
**Cell Lines**				
**Study**	**Disease**	**Sample**	**miRNAs**	**Findings**
Chiba et al. (2009) [83]	Abnormal BSM contraction	BSM cells, bronchial tissues of BALB/c mice	miR-133a	Up-regulation of RhoA when endogenous miR-133a function inhibited by its antagomir in hBSMCs (*p* < 0.05). No effect (*p* > 0.05) of miR-133b and let-7a antagomirs
Pandit et al. (2010) [84]	Pulmonary Fibrosis	Animals: mice models. Cells: 10 Idiopathic Pulmonary Fibrosis and 10 control tissues	Let-7d	Let-7d antagomir decreased expression of CDH1 and TJP1, and increased COL1A1 and HMGA2 expression in the lungs (*p* < 0.05)
Bhattacharyya et al. (2011) [29]	Cystic Fibrosis	Lung epithelial cells	miR-155	Antagomir-155 in CF cells down-regulates miR-155 expression by 85%; IL-8 mRNA levels decreased of 70% and IL-8 protein levels by 11-fold
Chatterjee et al. (2014) [41]	Lung cell dysfunction	HLMECs, HUVECs	miR-147b	MiR-147b antagomir increased total and cell surface expression of ADAM15 in endothelial cells (*p* < 0.05)
Fabbri et al. (2014) [43]	Cystic Fibrosis	CF bronchial epithelial IB3-1 cells infected by Pseudomonas aeruginosa	miR-93	IL-8 up-regulation in uninfected cells treated with antagomiR-93 (*p* < 0.01)
Zhang et al. (2015) [52]	Various disorders	Animals: BALB/c mice. Cells: 4T1 murine breast cancer cells	miR-10b	Antagomir-10b and PTX delivered by D-Lip delays the growth of 4T1 tumors and reduce the lung metastases; up-regulated Hoxd10 in tumors (*p* < 0.01)
Zhou et al. (2015) [54]	Influenza	Animals: six groups of mice (five mice per group), including control group. Cells: MDCK cells	miR-2911	Inhibitory effect of HS decoction on viral replication abolished by anti-miR2911 (*p* < 0.05)
Ge et al. (2016) [82]	Lung fibrosis	Humans: bronchial epithelia from lung transplant patients. Cells: primary fibroblasts from human lung explants	miR-323a-3p	Antagomirs for miR-323a-3p augment murine lung fibrosis after bleomycin injury (*p* < 0.05)
Podsiad et al. (2015) [31]	Pneumonia	Animals: wild-type C57BL/6 mice. Cells: human lung macrophages	miR-155	AntimiR-155 improved lung bacterial clearance by 4.2-fold compared with controls
Zhou et al. (2016) [86]	Systemic Lupus Erythematosus	Animals: C57BL/6J (B6) and B6.Cg-Mir155^tm1.1Rsky/J^ mice. Cells: Hepa 1-6 cells	miR-155	Disease progression, reduced by 20% by in vivo silencing of miR-155 using antimiR-155
Bartoszewska et al. (2017) [36]	Hypoxia	Hypoxia-induced human airway epithelial cell lines Calu-3 and 16HBE14o-; normal primary bronchial epithelial cells	miR-200b	Manipulation of miRNA levels during normoxia and hypoxia by antagomirs increased CFTR mRNA levels (*p* < 0.05)
Ma et al. (2017) [71]	Hypoxia	Animals: adult male Wistar rats. Cells: PASMC cultured	miR-125a	miR-125a antagomir mimicked the hypoxic damage effects to mitochondrial homeostasis (*p* < 0.05)
Morales et al. (2017) [87]	SARS-CoV	Animals: Female mice. Cells: mouse delayed brain tumor cells expressing the murine SARS-CoV receptor ACE2	svRNA-nsp3.1, svRNA-nsp3.2, svRNA-N, miR-877	Antagomirs reduced partially (svRNA-nsp3.1), or totally (svRNA-nsp3.2, svRNA-N, miR-877), the luciferase activity
Shentu et al. (2017) [26]	Lung fibrosis	Human bone marrow-derived Mesenchymal Stem Cells	miR-199a/b-3p, 21-5p, 630, 22-3p, 196a-5p, 199b-5p, 34a-5p, and 148a-3p	AntagomiR-630 abrogated the effect of extracellular vesicles on CDH2 expression (*p* < 0.05)
Zhou et al. (2017) [88]	Influenza	Animals: beagles. Cells: MDCK cells	cfa-miR-143	Anti-cfa-miR-143 caused upregulation of Igfbp5 in CIV-infected MDCK cells
Sharma et al. (2018) [58]	HIV infection and substance abuse	Human monocyte derived macrophages, HPASMCs	miR-130a	Transfection of HPASMCs with antagomir-130a ameliorated the extracellular vesicles-induced effect (*p* < 0.001)
Yuan et al. (2018) [69]	Tuberculosis	Fifty patients, 20 controls. Monocytes isolated from peripheral blood mononuclear cells	miR-196b-5p	Antagomir-196b-5p promoted Bacillus Calmette–Guérin uptake in MDMs or differentiated U937 cells (*p* < 0.05)
Tamgue et al. (2019) [89]	Tuberculosis	Bone marrow-derived macrophages generated from male BALB/c mice	miR-143, miR-365	Antagomirs for miR-143 and miR-365 decreased the intracellular growth of Mtb HN878, reduced the production of IL-6 (*p* < 0.001) and CCL5 (*p* < 0.01 for miR-143, *p* < 0.05 for miR-365), and promoted the apoptotic death of Mtb HN878-infected BMDMs (*p* < 0.01 for miR-143, *p* < 0.05 for miR-365)
Zhang et al. (2019) [90]	Influenza	Animals: C57BL/6 mice. Cells: human pulmonary epithelial cell line A549	miR-146a	Downregulation of miR-146a inhibits Influenza A Virus replication by enhancing type I IFN response through TRAF6 in vitro and in vivo (*p* < 0.01)

## References

[1] Krol J., Loedige I., Filipowicz W. (2010). The widespread regulation of microrna biogenesis, function and decay. Nat. Rev. Genet..

[2] Bartel D.P. (2018). Metazoan micrornas. Cell.

[3] Gambari R., Brognara E., Spandidos D.A., Fabbri E. (2016). Targeting oncomirnas and mimicking tumor suppressor mirnas: Nuew trends in the development of mirna therapeutic strategies in oncology (review). Int. J. Oncol..

[4] Nguyen D.D., Chang S. (2017). Development of novel therapeutic agents by inhibition of oncogenic micrornas. Int. J. Mol. Sci..

[5] Jadideslam G., Ansarin K., Sakhinia E., Babaloo Z., Abhari A., Ghahremanzadeh K., Khalili M., Radmehr R., Kabbazi A. (2019). Diagnostic biomarker and therapeutic target applications of mir-326 in cancers: A systematic review. J. Cell Physiol..

[6] Yoo B.H., Bochkareva E., Bochkarev A., Mou T.C., Gray D.M. (2004). 2’-o-methyl-modified phosphorothioate antisense oligonucleotides have reduced non-specific effects in vitro. Nucleic Acids Res..

[7] Krutzfeldt J., Rajewsky N., Braich R., Rajeev K.G., Tuschl T., Manoharan M., Stoffel M. (2005). Silencing of micrornas in vivo with ’antagomirs’. Nature.

[8] Lee S.H., Jung Y.D., Choi Y.S., Lee Y.M. (2015). Targeting of runx3 by mir-130a and mir-495 cooperatively increases cell proliferation and tumor angiogenesis in gastric cancer cells. Oncotarget.

[9] Brognara E., Fabbri E., Montagner G., Gasparello J., Manicardi A., Corradini R., Bianchi N., Finotti A., Breveglieri G., Borgatti M. (2016). High levels of apoptosis are induced in human glioma cell lines by co-administration of peptide nucleic acids targeting mir-221 and mir-222. Int. J. Oncol..

[10] Liang Z., Ahn J., Guo D., Votaw J.R., Shim H. (2013). Microrna-302 replacement therapy sensitizes breast cancer cells to ionizing radiation. Pharm. Res..

[11] Pace E., Di Vincenzo S., Di Salvo E., Genovese S., Dino P., Sangiorgi C., Ferraro M., Gangemi S. (2019). Mir-21 upregulation increases il-8 expression and tumorigenesis program in airway epithelial cells exposed to cigarette smoke. J. Cell Physiol..

[12] Shrine N., Guyatt A.L., Erzurumluoglu A.M., Jackson V.E., Hobbs B.D., Melbourne C.A., Batini C., Fawcett K.A., Song K., Sakornsakolpat P. (2019). New genetic signals for lung function highlight pathways and chronic obstructive pulmonary disease associations across multiple ancestries. Nat. Genet..

[13] Hobbs B.D., Tantisira K.G. (2019). Micrornas in copd: Small molecules with big potential. Eur. Respir. J..

[14] Faiz A., Steiling K., Roffel M.P., Postma D.S., Spira A., Lenburg M.E., Borggrewe M., Eijgenraam T.R., Jonker M.R., Koppelman G.H. (2019). Effect of long-term corticosteroid treatment on microrna and gene-expression profiles in copd. Eur. Respir. J..

[15] Heffler E., Allegra A., Pioggia G., Picardi G., Musolino C., Gangemi S. (2017). Microrna profiling in asthma: Potential biomarkers and therapeutic targets. Am. J. Respir. Cell Mol. Biol..

[16] Yu B., Yao L., Liu C., Tang L., Xing T. (2019). Upregulation of microrna16 alters the response to inhaled betaagonists in patients with asthma though modulating expression of adrb2. Mol. Med. Rep..

[17] Galie N., Humbert M., Vachiery J.L., Gibbs S., Lang I., Torbicki A., Simonneau G., Peacock A., Vonk Noordegraaf A., Beghetti M. (2016). 2015 esc/ers guidelines for the diagnosis and treatment of pulmonary hypertension: The joint task force for the diagnosis and treatment of pulmonary hypertension of the european society of cardiology (esc) and the european respiratory society (ers): Endorsed by: Association for european paediatric and congenital cardiology (aepc), international society for heart and lung transplantation (ishlt). Eur. Heart J..

[18] Wheeler A.P., Bernard G.R. (2007). Acute lung injury and the acute respiratory distress syndrome: A clinical review. Lancet.

[19] Zhou T., Garcia J.G., Zhang W. (2011). Integrating micrornas into a system biology approach to acute lung injury. Transl. Res..

[20] Wang Q.Z., Xu W., Habib N., Xu R. (2009). Potential uses of microrna in lung cancer diagnosis, prognosis, and therapy. Curr. Cancer Drug Targets.

[21] Yu S.L., Chen H.Y., Chang G.C., Chen C.Y., Chen H.W., Singh S., Cheng C.L., Yu C.J., Lee Y.C., Chen H.S. (2008). Microrna signature predicts survival and relapse in lung cancer. Cancer Cell.

[22] Janssen H.L., Reesink H.W., Lawitz E.J., Zeuzem S., Rodriguez-Torres M., Patel K., van der Meer A.J., Patick A.K., Chen A., Zhou Y. (2013). Treatment of hcv infection by targeting microrna. N. Engl. J. Med..

[23] Bouchie A. (2013). First microrna mimic enters clinic. Nat. Biotechnol..

[24] Collison A., Mattes J., Plank M., Foster P.S. (2011). Inhibition of house dust mite-induced allergic airways disease by antagonism of microrna-145 is comparable to glucocorticoid treatment. J. Allergy Clin. Immunol..

[25] Pullamsetti S.S., Doebele C., Fischer A., Savai R., Kojonazarov B., Dahal B.K., Ghofrani H.A., Weissmann N., Grimminger F., Bonauer A. (2012). Inhibition of microrna-17 improves lung and heart function in experimental pulmonary hypertension. Am. J. Respir. Crit. Care Med..

[26] Shentu T.P., Huang T.S., Cernelc-Kohan M., Chan J., Wong S.S., Espinoza C.R., Tan C., Gramaglia I., van der Heyde H., Chien S. (2017). Thy-1 dependent uptake of mesenchymal stem cell-derived extracellular vesicles blocks myofibroblastic differentiation. Sci. Rep..

[27] Kim R.Y., Horvat J.C., Pinkerton J.W., Starkey M.R., Essilfie A.T., Mayall J.R., Nair P.M., Hansbro N.G., Jones B., Haw T.J. (2017). Microrna-21 drives severe, steroid-insensitive experimental asthma by amplifying phosphoinositide 3-kinase-mediated suppression of histone deacetylase 2. J. Allergy Clin. Immunol..

[28] Li J.W., Wei L., Han Z., Chen Z. (2019). Mesenchymal stromal cells-derived exosomes alleviate ischemia/reperfusion injury in mouse lung by transporting anti-apoptotic mir-21-5p. Eur. J. Pharmacol..

[29] Bhattacharyya S., Balakathiresan N.S., Dalgard C., Gutti U., Armistead D., Jozwik C., Srivastava M., Pollard H.B., Biswas R. (2011). Elevated mir-155 promotes inflammation in cystic fibrosis by driving hyperexpression of interleukin-8. J. Biol. Chem..

[30] Plank M.W., Maltby S., Tay H.L., Stewart J., Eyers F., Hansbro P.M., Foster P.S. (2015). Microrna expression is altered in an ovalbumin-induced asthma model and targeting mir-155 with antagomirs reveals cellular specificity. PLoS ONE.

[31] Podsiad A., Standiford T.J., Ballinger M.N., Eakin R., Park P., Kunkel S.L., Moore B.B., Bhan U. (2016). Microrna-155 regulates host immune response to postviral bacterial pneumonia via il-23/il-17 pathway. Am. J. Physiol. Lung Cell Mol. Physiol..

[32] Yuan Z., Syed M., Panchal D., Joo M., Bedi C., Lim S., Onyuksel H., Rubinstein I., Colonna M., Sadikot R.T. (2016). Trem-1-accentuated lung injury via mir-155 is inhibited by lp17 nanomedicine. Am. J. Physiol. Lung Cell Mol. Physiol..

[33] He S., Li Z., Yu Y., Zeng Q., Cheng Y., Ji W., Xia W., Lu S. (2019). Exosomal mir-499a-5p promotes cell proliferation, migration and emt via mtor signaling pathway in lung adenocarcinoma. Exp. Cell Res..

[34] Liu M., Wang Z., Yang S., Zhang W., He S., Hu C., Zhu H., Quan L., Bai J., Xu N. (2011). Tnf-alpha is a novel target of mir-19a. Int. J. Oncol..

[35] Lee H.Y., Lee H.Y., Choi J.Y., Hur J., Kim I.K., Kim Y.K., Kang J.Y., Lee S.Y. (2017). Inhibition of microrna-21 by an antagomir ameliorates allergic inflammation in a mouse model of asthma. Exp. Lung Res..

[36] Bartoszewska S., Kamysz W., Jakiela B., Sanak M., Kroliczewski J., Bebok Z., Bartoszewski R., Collawn J.F. (2017). Mir-200b downregulates cftr during hypoxia in human lung epithelial cells. Cell Mol. Biol. Lett..

[37] Baker J.R., Vuppusetty C., Colley T., Papaioannou A.I., Fenwick P., Donnelly L., Ito K., Barnes P.J. (2016). Oxidative stress dependent microrna-34a activation via pi3kalpha reduces the expression of sirtuin-1 and sirtuin-6 in epithelial cells. Sci. Rep..

[38] Baker J.R., Vuppusetty C., Colley T., Hassibi S., Fenwick P.S., Donnelly L.E., Ito K., Barnes P.J. (2019). Microrna-570 is a novel regulator of cellular senescence and inflammaging. FASEB J..

[39] Brock M., Samillan V.J., Trenkmann M., Schwarzwald C., Ulrich S., Gay R.E., Gassmann M., Ostergaard L., Gay S., Speich R. (2014). Antagomir directed against mir-20a restores functional bmpr2 signalling and prevents vascular remodelling in hypoxia-induced pulmonary hypertension. Eur. Heart J..

[40] Cha S.T., Chen P.S., Johansson G., Chu C.Y., Wang M.Y., Jeng Y.M., Yu S.L., Chen J.S., Chang K.J., Jee S.H. (2010). Microrna-519c suppresses hypoxia-inducible factor-1alpha expression and tumor angiogenesis. Cancer Res..

[41] Chatterjee V., Beard R.S., Reynolds J.J., Haines R., Guo M., Rubin M., Guido J., Wu M.H., Yuan S.Y. (2014). Microrna-147b regulates vascular endothelial barrier function by targeting adam15 expression. PLoS ONE.

[42] Chiu K.L., Kuo T.T., Kuok Q.Y., Lin Y.S., Hua C.H., Lin C.Y., Su P.Y., Lai L.C., Sher Y.P. (2015). Adam9 enhances cdcp1 protein expression by suppressing mir-218 for lung tumor metastasis. Sci. Rep..

[43] Fabbri E., Borgatti M., Montagner G., Bianchi N., Finotti A., Lampronti I., Bezzerri V., Dechecchi M.C., Cabrini G., Gambari R. (2014). Expression of microrna-93 and interleukin-8 during pseudomonas aeruginosa-mediated induction of proinflammatory responses. Am. J. Respir. Cell Mol. Biol..

[44] Fehl J., Pozarska A., Nardiello C., Rath P., Surate Solaligue D.E., Vadasz I., Mayer K., Herold S., Seeger W., Morty R.E. (2019). Control interventions can impact alveolarization and the transcriptome in developing mouse lungs. Anat. Rec. (Hoboken).

[45] Fu L., Zhu P., Qi S., Li C., Zhao K. (2018). Microrna-92a antagonism attenuates lipopolysaccharide (lps)-induced pulmonary inflammation and injury in mice through suppressing the pten/akt/nf-kappab signaling pathway. Biomed. Pharmacother..

[46] Guo L., Liu Y., Bai Y., Sun Y., Xiao F., Guo Y. (2010). Gene expression profiling of drug-resistant small cell lung cancer cells by combining microrna and cdna expression analysis. Eur. J. Cancer.

[47] Huang Y., Huang L., Zhu G., Pei Z., Zhang W. (2019). Downregulated microrna-27b attenuates lipopolysaccharide-induced acute lung injury via activation of nf-e2-related factor 2 and inhibition of nuclear factor kappab signaling pathway. J. Cell Physiol..

[48] Incoronato M., Garofalo M., Urso L., Romano G., Quintavalle C., Zanca C., Iaboni M., Nuovo G., Croce C.M., Condorelli G. (2010). Mir-212 increases tumor necrosis factor-related apoptosis-inducing ligand sensitivity in non-small cell lung cancer by targeting the antiapoptotic protein ped. Cancer Res..

[49] Jiang C., Guo Y., Yu H., Lu S., Meng L. (2019). Pleiotropic microrna-21 in pulmonary remodeling: Novel insights for molecular mechanism and present advancements. Allergy Asthma Clin. Immunol..

[50] Li J.J., Tay H.L., Maltby S., Xiang Y., Eyers F., Hatchwell L., Zhou H., Toop H.D., Morris J.C., Nair P. (2015). Microrna-9 regulates steroid-resistant airway hyperresponsiveness by reducing protein phosphatase 2a activity. J. Allergy Clin. Immunol..

[51] Mondejar-Parreno G., Callejo M., Barreira B., Morales-Cano D., Esquivel-Ruiz S., Moreno L., Cogolludo A., Perez-Vizcaino F. (2019). Mir-1 is increased in pulmonary hypertension and downregulates kv1.5 channels in rat pulmonary arteries. J. Physiol..

[52] Zhang Q., Ran R., Zhang L., Liu Y., Mei L., Zhang Z., Gao H., He Q. (2015). Simultaneous delivery of therapeutic antagomirs with paclitaxel for the management of metastatic tumors by a ph-responsive anti-microbial peptide-mediated liposomal delivery system. J. Control. Release.

[53] Zhang Y., Li M., Hu C. (2018). Exosomal transfer of mir-214 mediates gefitinib resistance in non-small cell lung cancer. Biochem. Biophys. Res. Commun..

[54] Zhou Z., Li X., Liu J., Dong L., Chen Q., Liu J., Kong H., Zhang Q., Qi X., Hou D. (2015). Honeysuckle-encoded atypical microrna2911 directly targets influenza a viruses. Cell Res..

[55] Zhu Q., Zang Q., Jiang Z.M. (2018). Enhanced expression of non coding mir 92a expression is implicated in the development of lung cancer. Eur. Rev. Med. Pharmacol. Sci..

[56] Rosenberger C.M., Podyminogin R.L., Navarro G., Zhao G.W., Askovich P.S., Weiss M.J., Aderem A. (2012). Mir-451 regulates dendritic cell cytokine responses to influenza infection. J. Immunol..

[57] Sharma S., Umar S., Centala A., Eghbali M. (2015). Role of mir206 in genistein-induced rescue of pulmonary hypertension in monocrotaline model. J. Appl. Physiol. (1985).

[58] Sharma H., Chinnappan M., Agarwal S., Dalvi P., Gunewardena S., O’Brien-Ladner A., Dhillon N.K. (2018). Macrophage-derived extracellular vesicles mediate smooth muscle hyperplasia: Role of altered mirna cargo in response to hiv infection and substance abuse. FASEB J..

[59] Shi Y., Liu C., Liu X., Tang D.G., Wang J. (2014). The microrna mir-34a inhibits non-small cell lung cancer (nsclc) growth and the cd44hi stem-like nsclc cells. PLoS ONE.

[60] Silveyra P., DiAngelo S.L., Floros J. (2014). An 11-nt sequence polymorphism at the 3’utr of human sftpa1 and sftpa2 gene variants differentially affect gene expression levels and mirna regulation in cell culture. Am. J. Physiol. Lung Cell Mol. Physiol..

[61] Sun B., Yang N., Jiang Y., Zhang H., Hou C., Ji C., Liu Y., Zuo P. (2015). Antagomir-1290 suppresses cd133(+) cells in non-small cell lung cancer by targeting fyn-related src family tyrosine kinase. Tumour Biol..

[62] Sun C.C., Li S.J., Yuan Z.P., Li D.J. (2016). Microrna-346 facilitates cell growth and metastasis, and suppresses cell apoptosis in human non-small cell lung cancer by regulation of xpc/erk/snail/e-cadherin pathway. Aging (Albany NY).

[63] Vera O., Jimenez J., Pernia O., Rodriguez-Antolin C., Rodriguez C., Sanchez Cabo F., Soto J., Rosas R., Lopez-Magallon S., Esteban Rodriguez I. (2017). DNA methylation of mir-7 is a mechanism involved in platinum response through mafg overexpression in cancer cells. Theranostics.

[64] Wu T., Chen W., Kong D., Li X., Lu H., Liu S., Wang J., Du L., Kong Q., Huang X. (2015). Mir-25 targets the modulator of apoptosis 1 gene in lung cancer. Carcinogenesis.

[65] Wu C., Xu B., Zhou Y., Ji M., Zhang D., Jiang J., Wu C. (2016). Correlation between serum il-1beta and mir-144-3p as well as their prognostic values in luad and lusc patients. Oncotarget.

[66] Wu L., Pu X., Wang Q., Cao J., Xu F., Xu L.I., Li K. (2016). Mir-96 induces cisplatin chemoresistance in non-small cell lung cancer cells by downregulating samd9. Oncol. Lett..

[67] Wu Y., He H., Ding Y., Liu S., Zhang D., Wang J., Jiang H., Zhang D., Sun L., Ye R.D. (2018). Mk2 mediates macrophage activation and acute lung injury by regulating let-7e mirna. Am. J. Physiol. Lung Cell Mol. Physiol..

[68] Xu Z., Zhang C., Cheng L., Hu M., Tao H., Song L. (2014). The microrna mir-17 regulates lung foxa1 expression during lipopolysaccharide-induced acute lung injury. Biochem. Biophys Res. Commun..

[69] Yuan Y., Lin D., Feng L., Huang M., Yan H., Li Y., Chen Y., Lin B., Ma Y., Ye Z. (2018). Upregulation of mir-196b-5p attenuates bcg uptake via targeting socs3 and activating stat3 in macrophages from patients with long-term cigarette smoking-related active pulmonary tuberculosis. J. Transl. Med..

[70] Xie Z., Chen W., Chen Y., Wang X., Gao W., Liu Y. (2017). Mir-768-3p is involved in the proliferation, invasion and migration of non-small cell lung carcinomas. Int. J. Oncol..

[71] Ma C., Zhang C., Ma M., Zhang L., Zhang L., Zhang F., Chen Y., Cao F., Li M., Wang G. (2017). Mir-125a regulates mitochondrial homeostasis through targeting mitofusin 1 to control hypoxic pulmonary vascular remodeling. J. Mol. Med. (Berl).

[72] Zhou M., Hara H., Dai Y., Mou L., Cooper D.K., Wu C., Cai Z. (2016). Circulating organ-specific micrornas serve as biomarkers in organ-specific diseases: Implications for organ allo- and xeno-transplantation. Int. J. Mol. Sci..

[73] Lin C.W., Chang Y.L., Chang Y.C., Lin J.C., Chen C.C., Pan S.H., Wu C.T., Chen H.Y., Yang S.C., Hong T.M. (2013). Microrna-135b promotes lung cancer metastasis by regulating multiple targets in the hippo pathway and lzts1. Nat. Commun..

[74] Mao G., Liu Y., Fang X., Liu Y., Fang L., Lin L., Liu X., Wang N. (2015). Tumor-derived microrna-494 promotes angiogenesis in non-small cell lung cancer. Angiogenesis.

[75] McCann J.V., Xiao L., Kim D.J., Khan O.F., Kowalski P.S., Anderson D.G., Pecot C.V., Azam S.H., Parker J.S., Tsai Y.S. (2019). Endothelial mir-30c suppresses tumor growth via inhibition of tgf-beta-induced serpine1. J. Clin. Investig..

[76] Hsu A.C., Parsons K., Moheimani F., Knight D.A., Hansbro P.M., Fujita T., Wark P.A. (2016). Impaired antiviral stress granule and ifn-beta enhanceosome formation enhances susceptibility to influenza infection in chronic obstructive pulmonary disease epithelium. Am. J. Respir. Cell Mol. Biol..

[77] Potus F., Malenfant S., Graydon C., Mainguy V., Tremblay E., Breuils-Bonnet S., Ribeiro F., Porlier A., Maltais F., Bonnet S. (2014). Impaired angiogenesis and peripheral muscle microcirculation loss contribute to exercise intolerance in pulmonary arterial hypertension. Am. J. Respir. Crit Care Med..

[78] Gubrij I.B., Pangle A.K., Pang L., Johnson L.G. (2016). Reversal of microrna dysregulation in an animal model of pulmonary hypertension. PLoS ONE.

[79] Xie W., Lu Q., Wang K., Lu J., Gu X., Zhu D., Liu F., Guo Z. (2018). Mir-34b-5p inhibition attenuates lung inflammation and apoptosis in an lps-induced acute lung injury mouse model by targeting progranulin. J. Cell Physiol..

[80] Adyshev D.M., Moldobaeva N., Mapes B., Elangovan V., Garcia J.G. (2013). Microrna regulation of nonmuscle myosin light chain kinase expression in human lung endothelium. Am. J. Respir. Cell Mol. Biol..

[81] Adyshev D.M., Elangovan V.R., Moldobaeva N., Mapes B., Sun X., Garcia J.G. (2014). Mechanical stress induces pre-b-cell colony-enhancing factor/nampt expression via epigenetic regulation by mir-374a and mir-568 in human lung endothelium. Am. J. Respir. Cell Mol. Biol..

[82] Ge L., Habiel D.M., Hansbro P.M., Kim R.Y., Gharib S.A., Edelman J.D., Konigshoff M., Parimon T., Brauer R., Huang Y. (2016). Mir-323a-3p regulates lung fibrosis by targeting multiple profibrotic pathways. JCI Insight.

[83] Chiba Y., Tanabe M., Goto K., Sakai H., Misawa M. (2009). Down-regulation of mir-133a contributes to up-regulation of rhoa in bronchial smooth muscle cells. Am. J. Respir. Crit Care Med..

[84] Pandit K.V., Corcoran D., Yousef H., Yarlagadda M., Tzouvelekis A., Gibson K.F., Konishi K., Yousem S.A., Singh M., Handley D. (2010). Inhibition and role of let-7d in idiopathic pulmonary fibrosis. Am. J. Respir. Crit. Care Med..

[85] Asquith M., Pasala S., Engelmann F., Haberthur K., Meyer C., Park B., Grant K.A., Messaoudi I. (2014). Chronic ethanol consumption modulates growth factor release, mucosal cytokine production, and microrna expression in nonhuman primates. Alcohol. Clin. Exp. Res..

[86] Zhou S., Wang Y., Meng Y., Xiao C., Liu Z., Brohawn P., Higgs B.W., Jallal B., Jia Q., Qu B. (2016). In vivo therapeutic success of microrna-155 antagomir in a mouse model of lupus alveolar hemorrhage. Arthritis Rheumatol..

[87] Morales L., Oliveros J.C., Fernandez-Delgado R., tenOever B.R., Enjuanes L., Sola I. (2017). Sars-cov-encoded small rnas contribute to infection-associated lung pathology. Cell Host Microbe.

[88] Zhou P., Tu L., Lin X., Hao X., Zheng Q., Zeng W., Zhang X., Zheng Y., Wang L., Li S. (2017). Cfa-mir-143 promotes apoptosis via the p53 pathway in canine influenza virus h3n2-infected cells. Viruses.

[89] Tamgue O., Gcanga L., Ozturk M., Whitehead L., Pillay S., Jacobs R., Roy S., Schmeier S., Davids M., Medvedeva Y.A. (2019). Differential targeting of c-maf, bach-1, and elmo-1 by microrna-143 and microrna-365 promotes the intracellular growth of mycobacterium tuberculosis in alternatively il-4/il-13 activated macrophages. Front. Immunol..

[90] Zhang F., Sun X., Zhu Y., Qin W. (2019). Downregulation of mir-146a inhibits influenza a virus replication by enhancing the type i interferon response in vitro and in vivo. Biomed. Pharmacother.

[91] Liao W., Dong J., Peh H.Y., Tan L.H., Lim K.S., Li L., Wong W.F. (2017). Oligonucleotide therapy for obstructive and restrictive respiratory diseases. Molecules.

[92] Mei D., Tan W.S.D., Wong W.S.F. (2019). Pharmacological strategies to regain steroid sensitivity in severe asthma and copd. Curr. Opin. Pharmacol..

[93] Testa U., Pelosi E., Castelli G., Labbaye C. (2017). Mir-146 and mir-155: Two key modulators of immune response and tumor development. Noncoding RNA.

[94] Sessa R., Hata A. (2013). Role of micrornas in lung development and pulmonary diseases. Pulm. Circ..

[95] Panwar B., Omenn G.S., Guan Y. (2017). Mirmine: A database of human mirna expression profiles. Bioinformatics.

[96] Zhang J.G., Wang J.J., Zhao F., Liu Q., Jiang K., Yang G.H. (2010). Microrna-21 (mir-21) represses tumor suppressor pten and promotes growth and invasion in non-small cell lung cancer (nsclc). Clin. Chim. Acta.

[97] Porteous M.K., Lee J.C. (2017). Primary graft dysfunction after lung transplantation. Clin. Chest Med..

[98] Liu G., Friggeri A., Yang Y., Milosevic J., Ding Q., Thannickal V.J., Kaminski N., Abraham E. (2010). Mir-21 mediates fibrogenic activation of pulmonary fibroblasts and lung fibrosis. J. Exp. Med..

[99] O’Connell R.M., Taganov K.D., Boldin M.P., Cheng G., Baltimore D. (2007). Microrna-155 is induced during the macrophage inflammatory response. Proc. Natl. Acad. Sci. USA.

[100] Comer B.S., Camoretti-Mercado B., Kogut P.C., Halayko A.J., Solway J., Gerthoffer W.T. (2015). Cyclooxygenase-2 and microrna-155 expression are elevated in asthmatic airway smooth muscle cells. Am. J. Respir. Cell Mol. Biol..

[101] Suojalehto H., Toskala E., Kilpelainen M., Majuri M.L., Mitts C., Lindstrom I., Puustinen A., Plosila T., Sipila J., Wolff H. (2013). Microrna profiles in nasal mucosa of patients with allergic and nonallergic rhinitis and asthma. Int. Forum Allergy Rhinol..

